# Development of the Feedback Quality Instrument: a guide for health professional educators in fostering learner-centred discussions

**DOI:** 10.1186/s12909-021-02722-8

**Published:** 2021-07-12

**Authors:** Christina E. Johnson, Jennifer L. Keating, Michelle Leech, Peter Congdon, Fiona Kent, Melanie K. Farlie, Elizabeth K. Molloy

**Affiliations:** 1 Department of Medical Education, Faculty of Medicine, Dentistry and Health Sciences, University of Melbourne, Melbourne, Victoria Australia; 2 Department of Physiotherapy, School of Primary and Allied Health Care, Faculty of Medicine, Nursing and Health Sciences, Monash University, Melbourne, Victoria Australia; 3 Faculty of Medicine, Nursing and Health Sciences, Monash University, Monash Health, Melbourne, Victoria Australia; 4 Royal Australian and New Zealand College of Psychiatrists, Melbourne, Victoria Australia; 5 Faculty of Medicine, Nursing and Health Sciences, Monash University, Melbourne, Victoria Australia

**Keywords:** Feedback, Effective feedback, Professional development, Health professional education, Workplace learning, Feedback instrument, Instrument development, Scale development, Psychometric evaluation, Factor analysis, MFRM

## Abstract

**Background:**

Face-to-face feedback plays an important role in health professionals’ workplace learning. The literature describes guiding principles regarding effective feedback but it is not clear how to enact these. We aimed to create a Feedback Quality Instrument (FQI), underpinned by a social constructivist perspective, to assist educators in collaborating with learners to support learner-centred feedback interactions. In earlier research, we developed a set of observable educator behaviours designed to promote beneficial learner outcomes, supported by published research and expert consensus. This research focused on analysing and refining this provisional instrument, to create the FQI ready-to-use.

**Methods:**

We collected videos of authentic face-to-face feedback discussions, involving educators (senior clinicians) and learners (clinicians or students), during routine clinical practice across a major metropolitan hospital network. Quantitative and qualitative analyses of the video data were used to refine the provisional instrument. Raters administered the provisional instrument to systematically analyse educators’ feedback practice seen in the videos. This enabled usability testing and resulted in ratings data for psychometric analysis involving multifaceted Rasch model analysis and exploratory factor analysis. Parallel qualitative research of the video transcripts focused on two under-researched areas, psychological safety and evaluative judgement, to provide practical insights for item refinement. The provisional instrument was revised, using an iterative process, incorporating findings from usability testing, psychometric testing and parallel qualitative research and foundational research.

**Results:**

Thirty-six videos involved diverse health professionals across medicine, nursing and physiotherapy. Administering the provisional instrument generated 174 data sets. Following refinements, the FQI contained 25 items, clustered into five domains characterising core concepts underpinning quality feedback: *set the scene*, *analyse performance*, *plan improvements*, *foster learner agency*, and *foster psychological safety*.

**Conclusions:**

The FQI describes practical, empirically-informed ways for educators to foster quality, learner-centred feedback discussions. The explicit descriptions offer guidance for educators and provide a foundation for the systematic analysis of the influence of specific educator behaviours on learner outcomes.

**Supplementary Information:**

The online version contains supplementary material available at 10.1186/s12909-021-02722-8.

## Background

In the health professions, face-to-face feedback plays a key role in workplace learning and can have a powerful impact on performance [1]. Common feedback approaches include more scheduled, comprehensive performance discussions, for example workplace-based assessments or end-of-attachment appraisals; or more brief impromptu comments or tips offered while delivering clinical care (often called ‘feedback on the run’). Recent feedback literature, underpinned by social constructivism, supports learner-centred feedback conversations in which learners actively participate, to gain knowledge they can use to enhance subsequent performance [2–5]. A performance discussion with an educator offers opportunities for a learner to advance their understanding of the key characteristics of the target clinical performance (‘where am I aiming for?’), how their own performance compares to this (‘where am I now?’), and work out what they can do to improve (‘how can I get closer?’) [6–9]. When learners and educators collaborate through an interactive dialogue, together they can generate new performance insights and strategies for improvement, individually tailored for the learner [10, 11].

However, the literature does not provide clear guidance on how to apply these principles in practice; that is, what can educators do to enact learner-centred feedback? Studies have identified a gap between recommended and observed practices. Frequently, educators dominate feedback episodes and learners play a passive role [12–14]. Learners report that often they do not find educators’ comments relevant, and struggle to understand or apply the information [15–18]. Educators typically undertake minimal training in feedback (when contrasted with the rigorous development of clinical skills) and report a lack confidence in their feedback skills [19–23]. It may be that, in the absence of alternative strategies, educators are simply repeating feedback rituals they experienced as students or using formulaic assessment rubrics, which are not designed with an *interactive process* in mind. Hence there is a need for new schemas that are structured to promote educator and learner collaboration during feedback interactions [24–26].

A number of feedback models have been described in health professions education literature [27–29]. These provide useful insights to assist educators’ feedback practice. Some were designed for specific contexts such as formal discussions regarding written performance assessments [30], experiential communication skills training [28], or debriefing in simulation-based education [29]. Many of these guiding models were developed based on expert opinion, focused literature reviews or theoretical perspectives (or combinations of these). A few have reported modifications based on testing, such as inter-rater reliability or usability testing [29–31].

Our research program is focused on assisting educators to facilitate high quality, learner-centred, feedback interactions in clinical practice. It is based on a social constructivist paradigm, in which people actively build and refine their mental schemas during interactions with others at work [11]. We have focused on the educator, as ‘one partner in the dance’, because educators typically have a major influence on feedback interactions and have a responsibility to promote rich learning opportunities [25, 32]. Our goal is to create an instrument, the Feedback Quality Instrument to guide educators in high quality learner-centred feedback, by describing specific behaviours considered to enhance learner outcomes. This could contribute to clarifying ‘what quality feedback looks like’ and enable further analysis of which feedback components have the greatest beneficial impact.

**Fig. 1:**
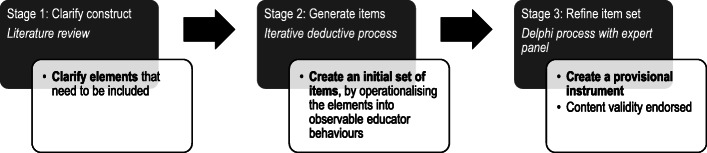
Development of the Feedback Quality Instrument: Completed Phase 1, Stages 1–3 to create a provisional feedback instrument

The development of the Feedback Quality Instrument is described in two phases. In Phase 1, a provisional instrument was created (previously published) [33] and in Phase 2, the focus of this article, the provisional instrument was analysed and refined [34, 35]. Phase 1 contained the following three stages (see Fig. 1):

Stage 1 - Clarifying the construct (i.e. constituents to be included in the instrument): an extensive review of the literature was conducted to identify discrete elements of an educator’s role considered to influence learner outcomes that were supported by empirical information. The review identified over 170 relevant articles across health professions education, education, business and psychology literature and included analyses of feedback observations, forms, surveys and interviews; feedback models; systematic reviews; consensus documents; and educational and psychological theories;

Stage 2 - Generating initial items: an iterative deductive process was used to convert the elements, identified in the literature review, into representative observable educator behaviour descriptions (items);

Stage 3 - Expert refinement of the initial item set: a Delphi process involving an expert panel led to consensus on a set of items with content validity.

**Fig. 2:**
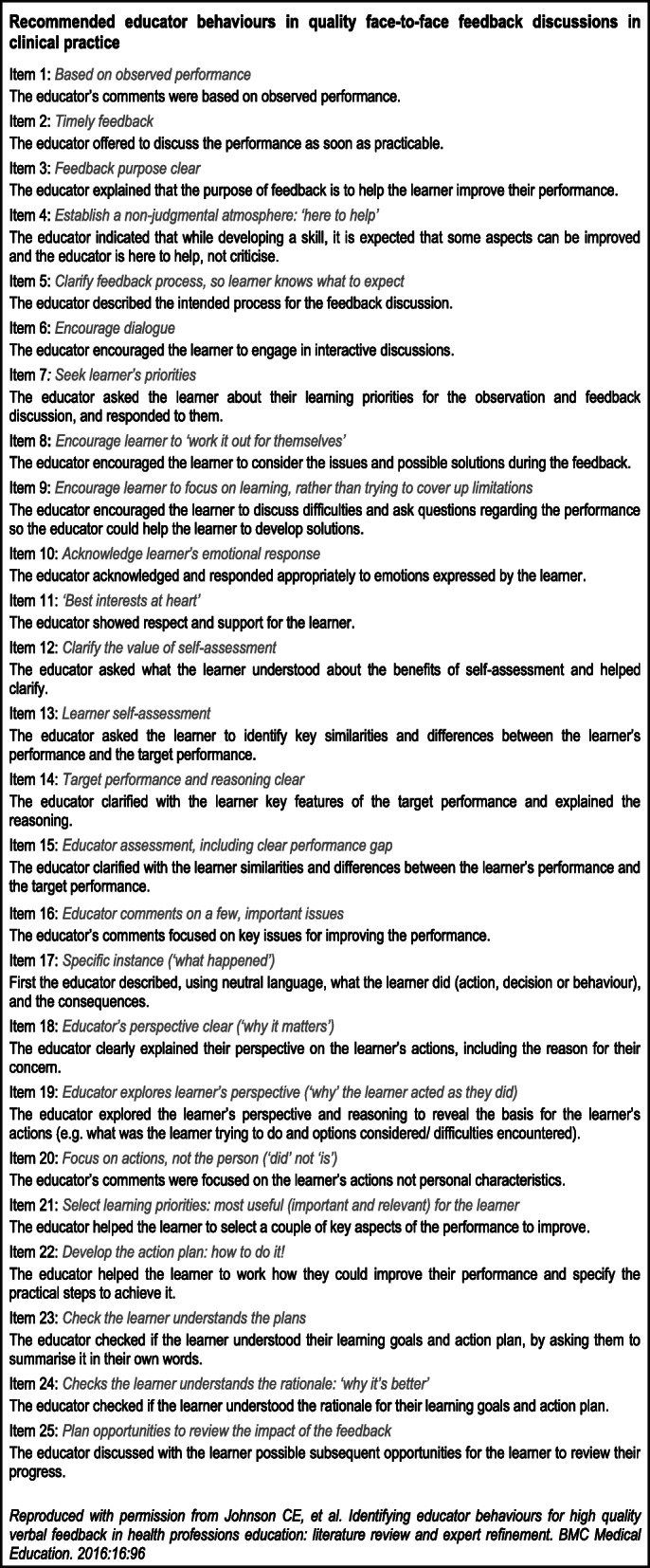
Set of items constituting a provisional feedback instrument

Hence Phase 1 resulted in a provisional instrument (reproduced in Fig. 2), incorporating a set of observable educator behaviours designed to foster learners’ engagement, motivation and capacity to improve [33].

The purpose of this current research, Phase 2, was to analyse and refine the provisional instrument, and present the Feedback Quality Instrument, validated and ready for use in clinical practice. For Phase 2, our research question was:

In what ways can the provisional instrument be refined, based on usability testing, psychometric analysis and parallel qualitative analyses of video data of authentic feedback interactions, to produce the Feedback Quality Instrument?

## Methods

### Research overview

**Fig. 3:**
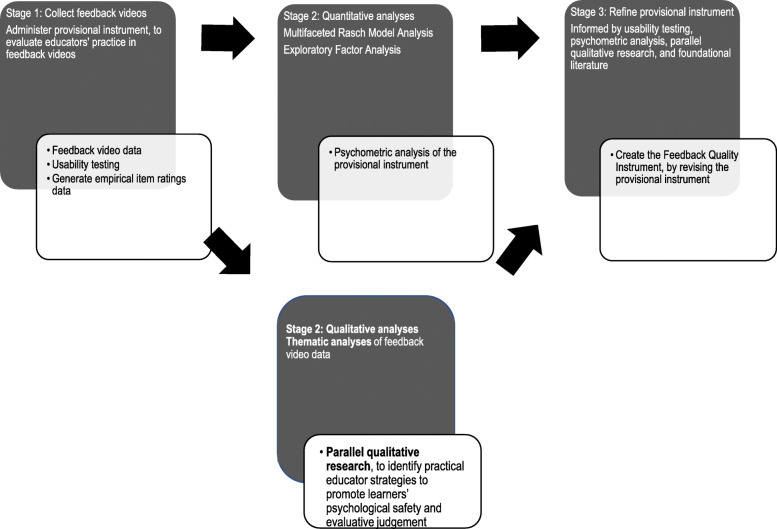
Development of the Feedback Quality Instrument: Phase 2: Testing, analysis and refinement of the provisional instrument to produce the Feedback Quality Instrument

This research used a multi-phased mixed methods design. Phase 1 developed a set of 25 items, representing a provisional feedback quality instrument, briefly summarised above and described in more detail elsewhere [33]. This article describes Phase 2 in which the provisional instrument was refined, based on quantitative and qualitative analysis of feedback discussions in clinical practice, to produce the Feedback Quality Instrument. Phase 2 involved three stages (see Fig. 3):

Stage 1 – Collecting feedback videos and administering the provisional instrument: Videos of authentic feedback discussions in routine clinical practice were collected. Then the provisional instrument was used to systematically evaluate educators’ practice seen in the feedback videos; this enabled usability testing and provided item ratings for psychometric analysis;

Stage 2 - Quantitative and qualitative analyses of video data to refine the provisional instrument: Psychometric testing of the item ratings data was conducted using Multifaceted Rasch Model (MFRM) analysis and exploratory factor analysis (EFA). Qualitative analyses of the video transcripts, reported in detail elsewhere, investigated two important but under-researched aspects of feedback, evaluative judgement [36] and psychological safety [37]. In particular, additional items were created for one instrument domain, *foster psychological safety*, as it was considered to be inadequately characterised following EFA analysis and a review of the latest literature did not reveal the practical information required.

Stage 3: Creating the Feedback Quality Instrument: the provisional instrument was revised based on usability testing, psychometric testing, qualitative research studies and underpinning research and theory (see Fig. 3).

Ethics approval was obtained from the health service (Reference 15,233 L) and the university human research ethics committees (Reference 2,015,001,338).

### Stage 1: collecting feedback videos and administering the provisional instrument

#### Collection of feedback videos

Videos of authentic scheduled feedback sessions were collected. To recruit participants for the feedback videos, first a diverse range of educators (supervising clinicians) across medicine, nursing and allied health in a major metropolitan teaching hospital network in Australia were invited to participate. When an educator consented, learners (students or clinicians) working with the educator at the time were invited to participate by the research team. Once both members in an educator-learner pair consented, they arranged to video themselves during the next face-to-face feedback session scheduled to discuss the learner’s performance in routine clinical practice. This methodology has been described in more detail previously [14].

#### Administering the provisional instrument

Raters administered the provisional instrument and compared educator behaviours seen in each feedback video with recommended educator behaviours (See Fig. 2 for the provisional instrument). Each item was rated as 0 = not seen, 1 = done somewhat, or 2 = done consistently. A pilot was conducted within the study to resolve preliminary problems using the instrument. This resulted in removal of *Item 2: The educator offered to discuss the performance as soon as practicable*, as this occurred before, not during, a feedback interaction. Subsequently all raters independently analysed all videos, which were presented in a random order devised using an online random number generator. Administration of the provisional instrument generated i) empirical item ratings data, subsequently used for psychometric analysis, and ii) usability analysis. (For more details regarding the raters and the pilot, see supplementary information: Section S1).

#### Usability analysis of the provisional instrument

While administering the provisional instrument, the rating team recorded comments regarding the usability of the instrument, items and rating scale, including both individual contemporaneous written comments during video analysis and two scheduled team telephone discussions, which were recorded [35]. (For more details, see supplementary information: Section S2).

### Stage 2a: quantitative analysis of feedback video data: psychometric analysis of the provisional instrument using item ratings data

To investigate the psychometric properties of the provisional instrument, the ratings data were used to conduct 1) multifaceted Rasch model analysis and 2) exploratory factor analysis.

#### Multifaceted Rasch model analysis (MFRMA)

The multifaceted Rasch model analysis examined how well the provisional instrument functioned as a measurement scale for estimating educators’ feedback proficiency, by analysing how closely the observed item ratings matched those expected by the model. The multifaceted Rasch model took account of the different aspects of the measurement system, including items, raters and rating scale categories, influencing the score (each called a ‘facet’) [38]. As the aim was to refine the provisional instrument, the analysis was primarily used to highlight items, raters or rating categories that showed substantial ‘misfit’ to the model, suggesting they may not usefully contribute, or may even degrade, the instrument’s performance as a measurement system, and may need modifying. A ‘person separation reliability’ level indicated how well the instrument discriminated between educators with different proficiency levels. The analysis created a linear interval scale, rather like ‘a feedback proficiency ruler’, based on the Likert ratings data from the provisional instrument. This was displayed on a ‘variable map’ that showed the spread of items (easy to difficult), participants (low to high proficiency) and raters (lenient to severe) on the same linear scale, enabling comparisons between them. (For more details on the MFRMA methods, see supplementary information: Section 3).

#### Exploratory factor analysis (EFA)

EFA is a common technique used to explore the characteristics of an instrument and guide its development [39–41] often in addition to Rasch analysis [42, 43]. The exploratory factor analysis, using principal components analysis and direct oblimin rotation, was conducted to identify clusters of closely inter-related items representing ‘factors’, indicating core concepts underlying ‘quality feedback proficiency’ [39, 44]. (For a comprehensive description of the EFA methods, see supplementary information – Section S4).

### Stage 2b: qualitative analysis of feedback video data

Qualitative analyses were conducted using thematic analysis of the video transcripts focusing on two particular aspects of feedback: psychological safety [37] and evaluative judgement, [36] described in previous publications. There is increasing interest concerning these important aspirations in quality feedback in the feedback literature but we found little practical guidance on how educators can collaborate with learners to promote them. Therefore, we conducted thematic analysis of the feedback video transcripts to identify how educators in our study had nurtured learners’ psychological safety and evaluative judgement during the feedback sessions, to enable revisions to the provisional instrument [45].

Psychological safety was defined by Edmondson as “a shared belief that the team is safe for interpersonal risk taking”, which creates “a sense of confidence that the team will not embarrass, reject or punish someone … due to mutual respect and trust” ([46] p354) Similar concepts discussed in the literature include ‘trust’ [47], the ‘educator-learner relationship’ [27, 48] the ‘educational alliance’ [49, 50] and creating a ‘safe container’ [51, 52]. When learners participate in learning conversations, they may expose their limitations by raising performance difficulties, explaining their reasoning or asking questions, which risks their professional reputation. At times learners choose to take this risk, in the hope of enhancing their skills and achieving their career goals. Hence it seems likely that learners’ sense of psychological safety will influence their level of involvement and vulnerability during feedback discussions.

Evaluative judgement was defined by Tai et al as “the capability to make decisions about the quality of work of self and others” [53]. Knowing ‘what good work looks like’ is a key skill underpinning life-long learning, as tacit standards need to be understood and applied in daily work [3, 54]. Feedback interactions provide valuable opportunities for learners to develop their evaluative judgement by analysing their performance in comparison with the desired performance. Educators can assist by encouraging learners’ self-assessment, clarifying key features of the desired performance and confirming the learner’s evaluation or explaining an alternative view, to help calibrate the learner’s judgement.

### Stage 3: refinement of the provisional instrument

The instrument and individual items were modified to better achieve the desirable criteria, previously established, that a) the instrument overall should achieve a comprehensive yet parsimonious set of items, that is, just enough items to sufficiently cover important discrete elements of an educator’s role in quality learner-centred feedback interactions across the full range of feedback proficiency; b) individual items should be generally applicable to verbal face-to-face feedback interactions, target a single distinct attribute, describe an observable educator behaviour, be unambiguous (phrasing clear and simple, so the meaning is easily and consistently understood without further explanation) and make sense with each rating category; c) the rating category options should be just sufficient to cover likely possibilities, and the phrasing of the rating categories should be consistent, clear and simple.

**Fig. 4:**
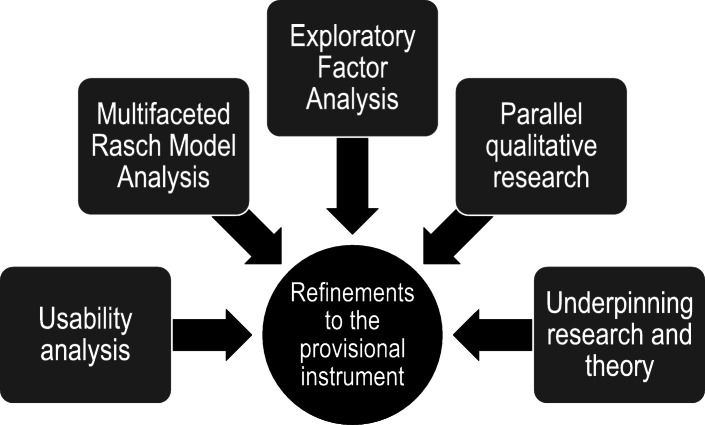
The multiple inputs that informed refinements to the provisional instrument, to create the Feedback Quality Instrument

Revisions to the provisional instrument were informed by 1) usability analysis, 2) psychometric analysis involving multifaceted Rasch model and exploratory factor analysis, 3) qualitative studies on psychological safety and evaluative judgement and 4) key theoretical principles that support learner-centred feedback, particularly relating to learning, motivation, psychological safety, evaluative judgement, and performance improvement (see Fig. 4). Modifications to items and the instrument overall were made using an iterative process (inductive and deductive) involving multiple rounds of revision and review based on all relevant considerations by a subgroup (CEJ, JLK, EKM), in consultation with the research team and key experts from our previous Delphi panel.

**Fig. 5:**
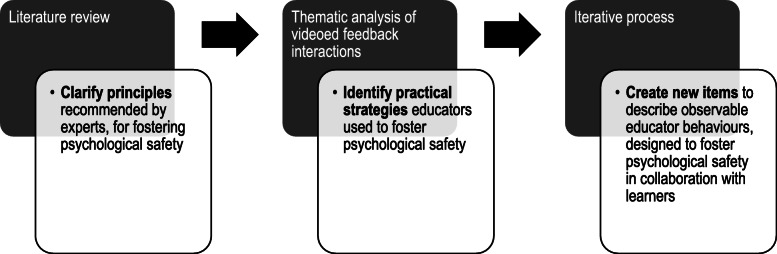
Process used to develop additional items for one domain, related to psychological safety, in the Feedback Quality Instrument

In particular, the EFA revealed factors, involving clusters of items, within quality feedback. During the instrument revision process, items were organised accordingly, to create domains in the Feedback Quality Instrument. If a factor was considered to be insufficiently characterised by those items, this triggered a process to create supplementary items. This decision was based on 1) the number of items. It is recommended a factor contain at least three items (although two items may comprise a factor if they are strongly inter-related with each other and relatively unrelated to other items) [41] and typically, complex concepts necessitate several items to elucidate and operationalise them [39]; and 2) a further review of relevant theory and research published in the literature, to identify relevant elements. Consequently, as explained in the results, the findings from the psychological safety study were used to create additional items in the relevant domain, in accordance with desirable item criteria described above, and using the same iterative process (see Fig. 5).

## Results

### Collecting feedback videos and administering the provisional instrument

#### Feedback videos and health professional participants

Thirty-six videos of scheduled feedback discussions during routine clinical practice were collected, involving educator-learner pairs across different health professions and specialities, experience levels and gender. In particular, there were 34 educators including 26 medical from every major speciality, 4 nursing and 4 physiotherapy health professionals. (For more details on the participants, see supplementary information: Section 5.1).

#### Using the provisional instrument to evaluate educators’ feedback practice

Each video was analysed by four to six raters, as unexpected time constraints prevented two researchers from analysing all of the videos (1 rater analysed 21/36 (58%) and 1 rater analysed 10/36 (28%)). This yielded 174 sets of ratings data. Missing data were uncommon (0.2% ratings missing). (For item ratings frequency data, see supplementary information: Section 5.2). Additional information including descriptive statistics of educators’ behaviours has been described elsewhere [14].

#### Usability analysis of the provisional instrument

Raters reported issues related to items 1, 7, 11, 12, 13, 17, 18, 19, such as overlapping items, ambiguous phrasing, restricted applicability or difficulty utilising rating categories, so these items were flagged for review. (For more details on usability analysis, see supplementary information: Section 5.3).

### Multifaceted Rasch model analysis

#### Item, rater and rating category analysis, and person separation reliability

In the MFRMA, items 5, 6, 8, 14, 15, 16 and 23 demonstrated misfit, so all these items were flagged for review, with a particular focus on items 5, 6, 14 and 23, which demonstrated misfit in the sensitivity analysis designed to isolate problems due to items alone, especially Item 5 that demonstrated more serious misfit.

Rater severity across the different raters was fairly similar except for Rater 2, whose ratings were more severe, indicated by severe misfit. Rater severity may be modified with training but consistency in rater severity is more important and MFRMA adjusts educator proficiency scores to take account of rater severity.

Rating category 1 (1 = done somewhat) showed misfit, so potential reasons for this were investigated. (For more details on item, rater severity, and rating category fit, see supplementary information: Section 6).

The person separation reliability was 0.95, which indicated the provisional instrument with multiple raters, could differentiate at least 4 levels of feedback proficiency amongst the educators.

#### Variable map

**Fig. 6:**
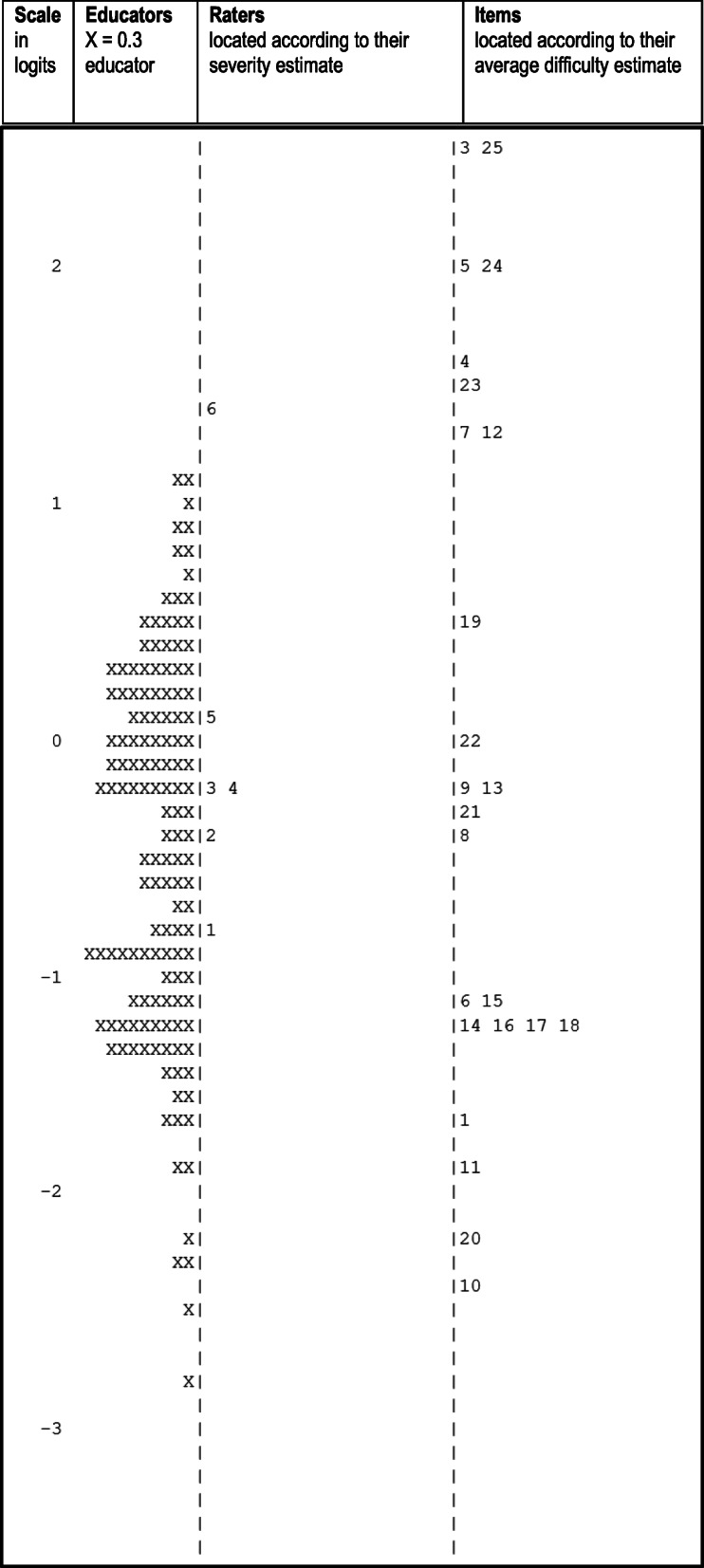
Variable map showing clinical educator proficiency, rater severity and item difficulty on the same interval scale. Footnote: Educators are shown as X = 0.3 to provide a slight distribution incorporating each educator’s estimate of their feedback proficiency and standard error

The variable map is presented in Fig. 6. From left to right, the variable map displays the linear interval scale (the ‘feedback proficiency ruler’), using ‘logits’ as the unit of measurement, and the distribution of educator feedback proficiency, rater severity and item difficulty on the same scale. The scale is set with the mean educator feedback proficiency estimate at zero logits. In particular, it can be seen that items and participants are reasonably distributed across the feedback proficiency range. (For more details on the variable map, see supplementary information: Section 6).

### Exploratory factor analysis

Exploratory factor analysis revealed five factors, represented by closely related item clusters, that constituted ‘quality feedback’. Four factors had multiple items that were strongly inter-related and theoretically aligned, which were named accordingly: *set the scene, analyse performance, plan improvement* and *foster learner agency*. The fifth factor only had two items but these were strongly inter-related and theoretically aligned, and it was named *foster psychological safety*. Items 5, 7 and 25 did not cluster strongly in any one factor, suggesting potential problems, so these were flagged for review. (For more details of the EFA results, see supplementary information: Section 7).

### Refinement of the provisional instrument

Multiple refinements were made to the provisional instrument, based on the results from the quantitative and qualitative analyses (see Fig. 4, and Table 1 for specific outcomes, typical reasons and potential actions arising from the usability, psychometric analysis and thematic analyses). The variable map from the MRFMA showed the spread of items across the range of feedback proficiency was acceptable with no substantial gaps, redundancy, ceiling or floor effects. The EFA identified item clusters, representing core concepts underlying quality feedback, so items in the instrument were regrouped accordingly. This provided a way to clarify the major domains and make it easier for users to understand the core concepts constituting quality feedback, instead of a large number of separate items.

**Table 1:** Analysis outcomes, typical reasons for those outcomes and subsequent potential actions to refine the provisional instrument, arising from usability analysis, exploratory factor analysis and multifaceted Rasch model analysis

	Analysis outcomes	Typical reasons	Potential actions
**Usability analysis**	**•Identify instrument problems**	• Not easy to use e.g. too many individual items or insufficient / complex instructions	• Find a way to simplify instrument administration e.g. group related items • Offer clear, useful and succinct instructions
		• Item gap	• Create new items to address gap
	**• Identify item problems**	• Items overlap	• Merge items
		• Item not generally applicable during a feedback interaction	• Remove or rephrase so item is generally applicable
		• Item phrasing: description of educator behaviour vague or not-observable	• Remove or rephrase so item clearly and simply describes pertinent observable behaviours
	**• Identify rating category problems**	• Too many rating categories, so hard to differentiate between them	• Reduce the number of rating categories
		• Rating category phrasing vague or not consistent across categories	• Rephrase rating category description so it is consistent, clear and simple
		• Middle rating category not applicable in some items	• Rephrase item so all rating categories are applicable
**Exploratory factor analysis**	**• Identify factors (core concepts) underlying quality feedback, represented by item clusters**	• Items in clusters are closely aligned i.e. all attributes of one concept	• Group items into instrument domains, and name accordingly
	**• Determine if each factor is adequately characterised, with sufficient items strongly aligned with it (3 items minimum, typically)**	• Insufficient items (e.g. only 2 items that strongly align)	• Create new items to describe observable behaviours that reflect that concept
	**• Identify items that do not align strongly with a single cluster**	• Item alignment split between 2 clusters (e.g. due to item phrasing or context)	• Remove or revise item, to better align with one cluster
		• Item does not strongly align with any cluster (e.g. due to item phrasing problems; item behaviour not sufficiently influential in the factor; or insufficient data)	• Remove or revise item, to align with one cluster
**Multifaceted Rasch model analysis**	**• Identify misfit shown by items, raters or rating category, which may distort the measurement system**	• Lack of consistent interpretation of item and application of rating category, due to: - item phrasing problems, so interpretation is variable - rating category problems, so application is variable • Insufficient data (if behaviour or rating category rarely employed)	Enhance consistency by • Removing or revising items and rating categories, according to desirable criteria • Using instrument manual and rater training
	**• Determine spread of items across range of ‘feedback proficiency’ (illustrated on the variable map)**	• Span with no items (gap)	• Create new items to address gap
		• Span with too many items (redundant items)	• Remove items to reduce redundancy

From the EFA, two items (items 10 and 11) constituted a fifth factor, *foster psychological safety.* It was decided that these items alone did not adequately characterise this important concept, so a process was initiated to create additional items. These new items, which described observable educator behaviours designed to foster psychological safety in collaboration with learners, were created by operationalising the findings from our qualitative study into psychological safety and related principles identified in the literature. Item development was performed by a subgroup (CEJ, JLK, EKM) using an iterative process, combining inductive and deductive reasoning, during multiple rounds of revision and review.

In addition, the findings from the qualitative analyses into evaluative judgement and psychological safety contributed to revising relevant items (for more details on the study findings, see supplementary information: Section S8).

Individual item modifications, based on inputs from all analyses, involved merging overlapping items, improving the phrasing of items (common revisions included making the description of pertinent observable behaviours more clear, simple and specific; generally applicable during feedback interactions; and make sense with each rating category) and adding succinct additional information to clarify further, if required. Details of the item refinements are outlined in detail in Appendix 1. The rating scale was revised to make the phrasing more consistent across rating categories. Subsequently, the instrument rating was: *Across the feedback session, how consistently did the educator do this? 0 = not done; 1 = done sometimes; 2 = done consistently.* For once off items, for example FQI item 1, if the educator demonstrated the behaviour as described in the item, this should be rated as 2 = done consistently.

### The Feedback Quality Instrument

**Fig. 7:**
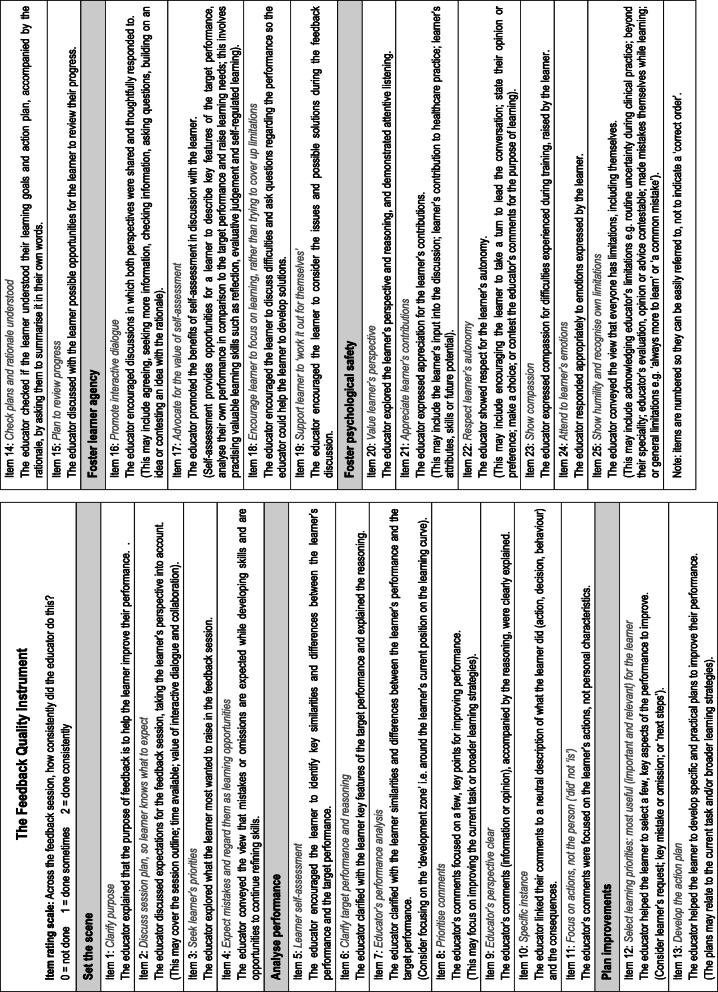
The Feedback Quality Instrument

**Fig. 8:**
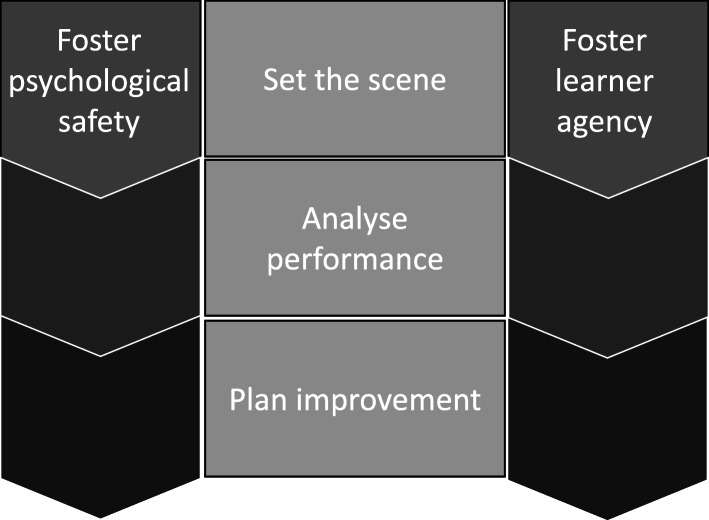
Schematic diagram showing the five domains, representing core concepts underpinning high quality feedback, within the Feedback Quality Instrument

On completion of this multi-phased mixed methods research process, incorporating empirical insights from the literature, usability analysis, psychometric analysis and qualitative studies into psychological safety and evaluative judgement, the Feedback Quality Instrument, ready for use, is presented in (see Figs. 7 and 8).

## Discussion

This research resulted in the creation of the Feedback Quality Instrument (FQI) (see Figs. 7 and 8) by refining a provisional feedback instrument, developed earlier [33]. To our knowledge, no other feedback instrument designed for clinical practice has undergone such a rigorous development process (see Figs. 1 and 3). The FQI clarifies how educators can work together with learners to foster high quality learner-centred feedback discussions in clinical practice. The items describe educator behaviours designed to engage learners in an interactive learning dialogue. This moves beyond tips focused on making educators’ input useful (e.g. timely, relevant, specific), to supporting learners to reveal difficulties, ask questions and refine ideas, so learners can enhance their understanding of their work, the required standards and instigate improvements. By attempting to explicitly characterise the educator’s role, we hope to ignite debate and research that leads to continuing refinements. We recognise that every feedback interaction needs to be customised, so the sequence or emphasis will vary depending on the individuals and the specific context.

Additionally, it is important to enhance the capacities of both educators and learners to effectively contribute to these conversations. We have chosen to focus on investigating the educator’s role in promoting beneficial learner outcomes and we recommend that readers consider complimentary work exploring ways to optimise the learner’s role, including proactively seeking and using feedback information [3, 53, 55–57].

The FQI contains five domains, three that occur somewhat sequentially, *set the scene, analyse performance, plan improvement,* and two that continue throughout the interaction, *foster psychological safety* and *foster learner agency* (see Figs. 7 and 8). The aim of the first domain, *set the scene* is to ‘start off on the right track’ by introducing important conditions for shaping the interaction from the beginning. Items in this domain express the educator’s intention to help the learner improve; an acceptance that mistakes or omissions are expected while developing skills, arising from a growth mindset, [58] and involve the learner in a discussion about expectations and learning priorities for the session. However in our feedback videos, a comprehensive introduction was rarely seen [14]. In simulation-based education, a ‘pre-brief’ routinely occurs to explain goals, expectations and plans for the session and to foster a ‘safe container’ [51]. Work in the area of doctor-patient communication has highlighted the value of involving patients in developing the agenda, to set up a collaborative consultation [59]. In contrast, when someone does not know what is going to happen and feel they have little control over it, this promotes anxiety. Excessive anxiety interferes with attention, processing information and memory, all of which are important operations for learning [60].

The next domain, *analyse performance,* focuses on the crucial step of assisting the learner to develop a clearer understanding of what the desired performance looks like and how their own performance compares with that [6, 48, 61]. Our qualitative analysis on evaluative judgement, published previously, contributed to revising these items in particular [36]. Items here highlight the value of clarifying key features of the target performance; grounding critique in specific examples to enhance understanding and credibility [15, 28, 50]; concentrating on ‘did’ not ‘is’ (otherwise, directing critique to personal identity offers limited prospects for change and risks strong emotional reactions) [62, 63] and prioritising discussion on a few points that are likely to be most useful for the learner, considering the learner’s priorities and skill trajectory [10, 48]. By endorsing aspects that the learner did correctly (or more correctly), the educator validates effective practice and confirms progress, which rewards effort, promotes intrinsic motivation and builds self-efficacy [64, 65]. Additionally, clarifying the performance gap helps focus learners’ attention on making improvements and paves the way for planning improvements [7, 66].

While *analyse performance* focuses on ‘making sense’, *plan improvements* deals with ‘making use’ of performance information [3, 55, 67]. Items in the *plan improvement* domain describe selecting important learning goals (such as addressing a significant error or responding to learner’s request) and designing effective improvement strategies, tailored to the individual. Yet, studies report that action plans are often omitted [14, 17, 68, 69]. Goal setting theory advocates that motivation, persistence and achievement are boosted when goals are clear and measurable (to determine progress), relevant and achievable (so effort is compensated by valuable results) and with a deadline (to focus attention) [64, 66].

The other two domains develop throughout a feedback conversation: *foster learner agency* and *foster psychological safety*. *Foster learner agency* incorporates themes of engagement, motivation and active learning [9, 64, 66, 70]. According to social constructivism, as learners and educators propose, consider and hone ideas by building on each other’s contributions, they co-create new insights and solutions [10, 11, 71]. The items describe ways to encourage learners to actively participate in interactive learning conversations; to focus on developing their skills by reflecting on their performance, raising problems, asking questions and generating ideas for improvement [9, 46, 70, 72]. When learners and educators critically analyse the learner’s performance together, this offers a valuable opportunity for learners to refine their mental schemas about both the current task and broader learning skills, particularly evaluative judgement [11, 36, 53]. Strategies to support active learning permeate the other domains. For example, items in *analyse performance* encourage learner self-assessment and prioritising topics for discussion to avoid cognitive overload; and items in *plan improvements* aim to ensure the learner understands the improvement strategy and rationale.

*Foster psychological safety* describes cultivating an environment in which *learner agency* can thrive. The importance of psychological safety stems from a learner’s moment-to-moment dilemma, where engaging in productive learning behaviours entails the risk of an adverse response [37]. For example, if a learner asks a potentially naïve question or contests an educator’s recommended strategy that the learner had tried to enact previously without success, this may expose undetected limitations in their knowledge and/or risk displeasing the educator [73, 74]. Research investigating learning and performance found that productive learning behaviours were common in ward teams with high psychological safety. These teams were characterised by three features: trust that co-workers had good intentions and were invested in each other’s success; interest, acceptance and care for each other as individuals; and respect for each other’s expertise [46, 75]. These traits could be summed up as ‘having someone’s best interests at heart’ and are embodied by collaboration. Based on principles identified in the literature and our own qualitative research study [37], FQI items depict ways educators can work with learners to nurture psychological safety; key themes include collaboration, respect, support and reducing the power gap [37, 47, 51, 52]. An educator can promote the partnership by creating sustained opportunities for a learner to share their thoughts regarding learning activities (e.g. reflections, concerns or opinions) and respond in ways that demonstrate appreciation, curiosity, respect and support (e.g. showing compassion or suggesting ideas for overcoming challenges) [76–78]. The inherent power imbalance between the defined roles of a supervisor/assessor and a learner may be moderated by educators demonstrating humility. In our feedback videos we saw educators acknowledge limitations in their own knowledge, assessment or advice; reveal difficulties they encountered during training [73, 79]; endorse life-long learning [72]; and appreciate the value of learners’ contributions [76, 77]. Again, these themes are embedded in items across all the other domains.

### Implications and future research

The FQI provides educators with a set of explicit behaviours designed to encourage a learner to collaborate in performance analysis and design of effective improvement strategies. Traditionally much advice for educators on feedback skills has contained principles such as ‘work as allies’, ‘build trust’ or ‘be learner centred’ but empirically-informed guidance on ‘what this looks like’ and how educators could help to cultivate these conditions, has been missing. We hope that by translating principles into actions and clearly articulating these standards, it will make it easier for educators to compare ‘their work’ (in this case, their contributions during feedback) with ‘what is expected’, just as learners do in trying to improve their clinical practice [6]. To support such professional development, we propose to create videos portraying feedback interactions to provide practical exemplars. These videos will involve actors performing fictional scenarios but informed by interactions in the authentic feedback videos, particularly demonstrations of good practice. The FQI offers a framework that educators can use when preparing for a feedback encounter, as a sensitising technique or afterwards, to analyse the encounter and trigger self-reflection. Clinicians could ask a colleague to observe their feedback practice, with learner consent, or instigate a ‘video club’ in which clinicians regularly discuss their own feedback practice videos [80]. In these situations, the critique could be stimulated by items on the FQI, rather than ‘gut feels’ about whether or not a feedback session was effective [81]. While watching videos (or role play) of feedback discussions, the FQI could be used to scrutinise interactions, match moments with corresponding items, select items they most wanted to discuss or to suggest improvements to observed practice. All these possibilities could be enhanced by involving learners as well as educators. This could assist everyday clinicians (educators and learners) in understanding each other’s perspectives and to ‘workshop’ various scenarios to gain expertise in promoting effective feedback interactions together. The FQI presents valuable opportunities to enhance both educator and learner feedback literacy and evaluative judgement within the health professions.

In addition, there may be potential for the FQI to be adapted for other contexts, such as higher education, to support a socio-constructivist feedback paradigm that focuses on educators and learners collaborating together [25, 26, 32].

We plan to undertake further testing of the FQI, including feasibility and ‘think aloud’ testing [82], and psychometric analysis using a larger sample, which may lead to further refinement. The FQI offers future opportunities to systematically analyse feedback to identify which educator behaviours, or combinations, have the greatest influence on learner outcomes. After all, the ultimate test for feedback quality is its effect [83]. This could identify a smaller number of the most useful behaviours, to create a ‘mini-FQI’ that is easier for everyday clinicians to adopt. Additionally, Rasch analysis of a finalised FQI could provide insights on a developmental trajectory in feedback proficiency, as Rasch analysis orders items (and therefore behaviours) from easiest to hardest. This could provide support for sequencing of educator training (analogous to a child learning to count, then add, then multiply during mathematical skills progression).

### Strengths and limitations of research

The strengths of this research lie in the rigorous development of the Feedback Quality Instrument. Phase 1, previously published, involved extensive literature searching for empirical evidence and Delphi processes with an expert panel to achieve consensus on a provisional feedback instrument [33]. Phase 2, detailed here, involved administering the provisional instrument to analyse routine feedback episodes with diverse health professionals, then refining it based on usability testing, psychometric analysis and parallel qualitative research on psychological safety and evaluative judgement.

There are a number of limitations to our research. Clinicians and students who volunteered to participate may not have been representative of supervising clinicians in general. Videoing feedback interactions may have influenced participant behaviour. Inconsistencies in observed ratings may be improved by item refinements and rater training using exemplars, calibration training and an instrument manual. The data set size was at the lower acceptable limit and a larger data set would enhance confidence in results from psychometric analysis. The FQI was designed in one country, involving multiple academics and clinicians across three states, and tested within one major healthcare network. Therefore, how applicable the instrument is to different countries and contexts is unknown.

## Conclusions

This study resulted in the Feedback Quality Instrument, ready-for-use in clinical practice. The FQI contains five domains portraying core concepts that constitute high quality feedback. Three domains occur sequentially, *set the scene*, *analyse performance* and *plan improvement* and two flow throughout a feedback encounter, *foster psychological safety* and *foster learner agency*. This instrument offers educators a set of explicit descriptions of useful behaviours to guide clinical workplace feedback. By orientating educators to what ‘learner-centred feedback looks like’, we hope it promotes conversations that help learners to develop.

## Supplementary Information

**Additional file 1.**

**Additional file 2.**

**Additional file 3.**

**Additional file 4.**

**Additional file 5.**

## Data Availability

The data sets used are contained in Appendix 2.
