# Supplementary material for: Development of the Feedback Quality Instrument: a guide for health professional educators in fostering learner-centred discussions
Source: BMC Med Educ. 2021 Jul 12;21:382. doi: 10.1186/s12909-021-02722-8 (PMC8276464; doi:10.1186/s12909-021-02722-8)
Supplement: Supplementary file 1 — Additional file 1. [file 12909_2021_2722_MOESM1_ESM.docx]

# Online supplementary information

# Methods

## S1 Administering the provisional instrument

The raters were all female health professionals with senior education and research roles (two doctors and four physiotherapists), including three with clinical roles in different units within the health service (two doctors and one physiotherapist).

As a pilot within this study, raters independently analysed three videos and then discussed utilising the provisional instrument and any related difficulties. Strategies to address the two problems identified were developed and implemented as follows. *Item 2: The educator offered to discuss the performance as soon as practicable* would occur prior, not during a feedback conversation, so it was removed from the instrument. Hence 24 items were scored and the total rating score range was 0 to 48. For *Item 10: The educator acknowledged and responded appropriately to emotions expressed by the learner,* it was decided to rate this as ‘2’ (done consistently) if either a) the learner displayed signs of emotion, including verbal indicators (e.g. defensive language); paraverbal indicators (e.g. slow monotone speech suggesting feeling disheartened); or nonverbal indicators (e.g. facial flushing suggesting embarrassment) and the educator responded appropriately, or b) if the learner’s demeanour throughout suggested a comfortable emotional equilibrium.

## S2 Usability analysis of the provisional instrument

Raters commented on ease of instrument use; item overlaps (when more than one item addressed similar educator behaviours) or gaps (when pertinent educator behaviours were not captured by items); item interpretation including phrasing that was too complex, could be interpreted in various ways, or did not effectively capture relevant observable behaviours (for example, if descriptions were either too nebulous or restrictive); and rating category interpretation and application [1].

## S3 Multifaceted Rasch Model analysis (MFRMA)

Height or weight can be directly measured using a ruler or scales respectively but ‘feedback proficiency’ cannot be directly measured as it is a psychosocial construct characterised by certain behaviours. The provisional feedback instrument contained items that described observable behaviours, rated using a Likert scale. The ‘distance’ between one rating category and the next on a Likert scale may not be uniform, and the items may vary in difficulty. Hence a total score, resulting from the sum of individual item scores, may not accurately reflect differences in overall proficiency levels between educators. Rasch models, based on item response theory, describe the characteristics of assessment instruments used to convert these type of ordinal data (i.e. Likert scale) into a linear interval performance measure [2-4]. In this case, the model computes the probability that an educator will demonstrate a specific behaviour described in an item in the feedback instrument, assuming it is solely determined by the educator’s proficiency and the difficulty of the behaviour described in the item. When an educator correctly demonstrates a specific behaviour, there is a high probability that they will correctly demonstrate behaviours described in all easier items. Items can be ranked from easiest to most difficult and this order remains consistent for educators with different proficiency levels. This allows ‘proficiency’ and ‘item difficulty’ to be represented on the same linear interval scale, which creates a measurement scale (or ‘ruler’) that estimates feedback proficiency. Several benefits arise from this. A linear interval scale allows differences between proficiency scores to be more accurately quantified (for example, when an educator improves their proficiency level following deliberate practice or to compare the proficiency levels of different educators) and typically, the data are more suitable for parametric analysis of the original ordinal data.

In this study, a Multifaceted Rasch Model was used, which took account of the influence of rater severity, item difficulty, rating categories and educator proficiency on the score (each called a ‘facet’) [5, 6]. As the main purpose was to refine the provisional instrument, the analysis focused on investigating how closely the observed item ratings matched those expected by the model. This was primarily assessed using model fit statistics; items with sufficient ‘misfit’ to the model may degrade the instrument’s function as a measurement scale. However, misfit only highlights items for review; it does not necessitate removal or revision. Item difficulty, educator proficiency and rater leniency were presented on the same linear interval scale (the ‘feedback quality ruler’) with ‘logits’ as the unit of measurement, illustrated in a variable map. This allowed comparisons between facets and components of facets (for example, individual items). In particular, the variable map provided information on whether the provisional instrument had sufficient coverage of items across the full range of feedback proficiency. The instrument’s power to discriminate between different proficiency levels demonstrated by educators was investigated using a separation reliability index. The MFRM analysis was conducted using ConQuest Generalised Item Response Modelling Software, Version 4, 2015 (Australian Council for Educational Research, Camberwell, Victoria).

### Model fit statistics

Fit statistics indicate how closely observed data match expected data generated by the MFRM model. Residuals are the difference between the observed and expected item ratings. The fit statistics included i) mean square of the residuals (MNSQ) with a 95% confidence interval and ii) a normalised equivalent (‘T’ statistic) [7, 8]. The MNSQ indicates the size of any misfit and the T statistic indicates the likelihood of misfit. Fit statistics are interpreted in a similar manner for all facets (items, raters and rating categories). A residual represents ‘effect on the instrument score, apart from the trait’ and denotes ‘noise’, as opposed to the ‘signal’ indicating the trait. In other words, ‘noise’ describes any contribution that detracts from the desired consistent interpretation of the item content and rating criteria across different contexts. Residuals can include an assortment of influences such as item phrasing; personal factors influencing participants’ performance or raters’ judgments, such as personal experience or fatigue; or different contexts.

The mean residual for all parameters is set to 1.0 and has a range from zero to infinity. The MNSQ also indicates the proportion of variance in observed responses from that expected by the model i.e. MNSQ of 1.20 indicates 20 percent more variance in responses than expected by the model and 0.70 is 30 percent less variance than expected by the model. In general, MNSQ values between 0.5 to 1.5 indicate acceptable fit. For clinical observations, MNSQ between 0.5 to 1.7 are deemed reasonable [8, 9]. High MNSQ values (known as underfit) indicate observed data are less predictable (i.e. more variable) than expected. MNSQ values between 1.5 and 2 are unproductive for measurement construction but not distorting; MNSQ values greater than 2 distort the instrument’s measurement system [9, 10]. T values greater than 2 indicate observed data are unlikely to fit the Rasch model (i.e. misfit is likely). On the other hand, low MNSQ values (known as overfit) below 0.5 indicate observed data are too predictable. T values greater than -2 indicate observed data are unlikely to fit the Rasch model. Overfit does not usefully contribute to the instrument’s measurement system but it does not distort it. The main concern is that overfit can artificially inflate the instrument’s separation reliability index [4].

‘Unweighted’ fit (sometimes called ‘outfit’) is sensitive to unexpected observed data involving participants with proficiency levels far away from the difficulty level of an item. Typical reasons for this include a careless mistake (e.g. when a participant with high overall proficiency does not demonstrate a behaviour described in an easy item) or subspecialty expertise (e.g. when a participant has a much higher proficiency in one particular area so they demonstrate a behaviour described in a difficult item, well above their overall proficiency level). ‘Weighted’ fit (sometimes called ‘infit’) corrects for this by giving more weight to item ratings for participants with proficiency levels close to the difficulty of that item. For example, when there is a large weighted fit value, this means the component (for example, an item) is not functioning well at the location where it is most discriminating, which is more serious. Typical reasons for this include the item targets an inconsequential behaviour, ambiguous item phrasing, or sparse data (making the result more sensitive to unexpected observations). On the other hand, when there is a large value for unweighted fit but not weighted fit, this is less serious as it is most likely due to idiosyncratic events.

In brief, misfit was used to identify specific facet components (items, raters or rating categories) for which observed data was more erratic than expected by the MFRM. Misfit was indicated by weighted MNSQ greater than 1.5 (suggesting items may not be usefully contributing the measurement system), particularly greater than 2 (suggesting items may be distorting the measurement system) and T greater than 2 (suggesting misfit was likely).

As the primary aim of this analysis was to refine the provisional instrument, a sensitivity analysis was conducted to better isolate potential problems due to the items themselves, by controlling for rater and rating scale problems that may have a substantial influence on item fit. When misfit was identified, this led to a thorough review of potential reasons, which informed consideration of subsequent modifications [9, 10].

#### Raters and rating scale fit statistics

Rater and rating category fit statistics provided information about how raters interpreted and applied items and rating categories. Rater underfit (MNSQ > 2 and T > 2) indicated raters who rated educators’ practice more erratically than expected by the Rasch model. Overfit (MNSQ less than 0.5 and T below -2) indicated raters with little variability in their ratings for educators across the range of feedback proficiency. Rating category fit statistics were used to identify inconsistent selection of a rating category at the expected proficiency level. Typical reasons for rating category misfit includes rating category problems, such as too many rating categories or vague descriptions of rating categories so it is hard to differentiate between them; or item phrasing problems that result in inconsistent selection or inapplicable rating categories.

### Separation reliability statistics

Separation reliability statistics for each facet provide an indication of discriminatory power. It is reported with a chi-square statistic (X^2^), to compare the distribution of components within each facet, the degrees of freedom (df) and a probability level (P). Separation reliability statistics are interpreted in a similar way as Cronbach’s alpha, with a range from 0 to 1 and larger values indicating greater consistency. The person separation reliability indicates the instrument’s ability to discriminate educators with different levels of feedback proficiency: 0.8 is acceptable and indicates ability to discriminate at least 2 different levels of feedback proficiency and 0.9 indicates ability to discriminate at least 4 different levels [11]. Multiple raters, as in this study, may result in higher reliability compared to single raters, as typically occurs in routine instrument administration.

### Variable map including item distribution

A variable map was used to show the spread of components within each facet on the same linear scale, including items (easy to difficult), participants (low to high proficiency) and raters (lenient to severe). In particular, the distribution of items was inspected. Each item can be considered as a ‘mark’ on the ‘feedback ruler’, so an ideal instrument would have items distributed across the full range of feedback quality, from very easy to very difficult items. If there were no items to measure the lowest or highest levels of proficiency, this could lead to ‘floor’ or ‘ceiling’ effects, respectively. There should be no substantial span without items; otherwise the instrument would be less able to precisely differentiate between proficiency levels, as it would not be generating as much information in that region. Conversely multiple items in a small span may lead to redundancy and unnecessary level of precision. Hence examination of the variable map enabled identification of gaps, redundancy and propensity to produce floor or ceiling effects.

## S4 Exploratory factor analysis

### Evaluating suitability of data for EFA

We assessed the suitability of the provisional instrument ratings data for EFA using Bartlett’s test of sphericity [12] (p <0.05 indicating suitability) and Kaiser-Meyer-Olkin measure of sampling adequacy [13] (a value greater than 0.6 is sufficient [14], with higher levels indicating increasingly compact correlation patterns with 0.71-0.8 described as ‘good’, 0.81-0.9 as ‘very good’ and over 0.9 as ‘excellent’) [15].

### Factor extraction

After determining that the data were suitable, principal components analysis (one EFA approach) was used to determine the most appropriate number of factors [16]. This involved balancing competing aims to account for as much variability in the data, explaining the inter-relationships between the items, with as few factors as possible. The first factor extracted explains the most variance within the data set and each subsequent factor extracted explains less variance. We selected the number of factors to analyse further by considering factor eigenvalues, the total variance extracted, the variance extracted by each factor, the scree plot and parallel analysis. An eigenvalue indicates the amount of total variance across all items explained by the factor, and typically only factors with eigenvalues greater than 1.0 are selected. Variance refers to the variability in observed results for items within a cluster that is explained by the underlying factor (that is, the underlying concept shared by the items). It is recommended that retained factors extract over 5% variance, with cumulative variance between 50 to 75% [17]. The scree plot is a graph that plots the eigenvalue against each factor in increasing numerical order [18]. It was used to identify factors that accounted for the greatest variance, positioned on the steep section of the curve before it flattened out. Parallel analysis was used to identify factors with eigenvalues greater than corresponding mean eigenvalues calculated from 100 random data sets of the same size, with the aim of excluding factors with eigenvalues no greater than those achieved in a random data set [19].

After selecting the number of factors to extract, factor rotation was conducted to assist with interpretation of item clusters. Factor rotation can offer alternative factor solutions, by rotating items in multidimensional space while maintaining inter-relationships. The aim is to produce a ‘simple structure’ in which each item strongly loads only onto one factor (typically, factor loading at least 0.4) [16]. Factor loading signifies the correlation between an item and a factor, which reflects how strongly the underlying concept (represented by the factor) influences the observed result for that item. Selecting the best arrangement of factors and item clusters involved making decisions based on both the statistical analyses (ideally each item strongly loads onto one factor only) and theoretical considerations (maintaining the theoretical foundations of the instrument) [17]. A direct oblimin technique was used; this ‘oblique approach’ makes no assumptions about relationships between factors. In addition, a varimax technique was used when factors were not closely related (when factor correlations were less than 0.3); this ‘orthogonal approach’ assumes the underlying constructs are independent. The varimax rotation aims to make the factors as distinct as possible, in which each factor only has items that strongly load onto it and no other factor, by amplifying the shared variance between the items and factors. Communality values provide information on the variance of each item explained by the factor, with values less than 0.3 indicating an item does not fit in well with the other items in the factor [16].

Once the best arrangement was selected, the factors were named according to the shared characteristic, indicated by the strongest loading items. The EFA was conducted using IBM SPSS Statistics for Windows, V25.0, 2017 (IBM Corp, Armonk, NY).

# Results

## S5 Collecting feedback videos and administering the provisional instrument

### S5.1 Feedback videos and health professional participants

The feedback session was typically videoed by setting up a computer or smart phone to record it. The discussion was based on the learner’s performance of a specific clinical task in 25 (69.4%) videos and across a clinical attachment in a mid/end-of-attachment appraisal in 11 (30.6%) videos. Most assessments were formative but some were summative contributions to longitudinal training evaluations. Participants included 34 educators and 35 learners (with two educators and one learner involved in two videos each). Educator characteristics included 18 (52.9%) women and 16 (47.1%) men; with 26 medical (six physicians, four surgeons, three anaesthetists, three emergency physicians, three psychiatrists, three obstetrician-gynaecologists, two radiologists, one paediatrician and one opthalmologist), 4 nursing and 4 physiotherapy health professionals. Experience levels included 14 (41.2%) educators with five years or less experience supervising learners, 11 (32.3%) with six to ten years, and 9 (26.5%) with more than ten years. The learner characteristics included 23 (65.7%) women and 12 (34.3%) men; in the same disciplines as their educators; and the following experience levels: 9 (25.7%) students, 9 (25.7%) clinicians with five years or less post-qualification, 15 (42.9%) clinicians with 6 years or more post-qualification and 2 (5.7%) senior clinicians.

#### S5.2 Item ratings frequency data

The frequency data for item ratings are shown in Table S1.

**Table S1 here**

#### S5.3 Usability analysis of the provisional instrument

Raters reported that it was demanding to rate 24 separate items in the provisional instrument (See Figure 2 in main article for item list). For specific items, they noted the following issues: Item 1 could be interpreted as ‘done’ in the context of a workplace based assessment; Items 1 and 17 targeted similar educator behaviours; Items 7, 12 and 13 included the phrase ‘the educator asked’ that either did or did not occur, so rating category 1 (done somewhat or sometimes) was inapplicable; Item 11 was too nebulous; and Items 17-19 were not generally applicable during a feedback discussion, as they had been designed as a subset of items to be used when an educator raised performance concerns but this did not occur in all interactions. Hence items 1, 7, 11, 12, 13, 17-19 were flagged for review.

## S6 Multifaceted Rasch Model analysis

### Item analysis: Item fit, including sensitivity analysis

Several items demonstrated misfit (weighted MNSQ > 1.5 and T > 2): items 5*, 6*, 8, 14, 15, 16* and 23 (* denotes MNSQ > 2, indicating more serious misfit). The detailed item fit data are presented in Table S2.

In the sensitivity analysis, designed to isolate problems due to items themselves, by reducing the effect of rater or rating category misfit, items 5*, 6, 14 and 23 demonstrated misfit (weighted MNSQ > 1.5 and T > 2; * denotes MNSQ > 2, indicating more serious misfit). The sensitivity analysis involved reducing the influence of rater and rating category problems on item fit by re-analysing data after i) removing data from raters 2 and 6, as rater 2 showed misfit and rater 6 was markedly severe (see additional information below under ‘rater analysis’ and Table S3) and ii) combining rating categories 1 and 2 to create one category ‘done, at least partially’, as rating category 1 showed misfit (see additional information below under ‘rater category analysis’ and Table S4). The provisional instrument had three rating categories: 0= ‘not seen’, 1 = ‘done somewhat or sometimes’, 2 = ‘done consistently’.

Therefore, all these items were flagged for review, particularly those items which demonstrated misfit in the sensitivity analysis and especially Item 5 which demonstrated more serious misfit (with weighted MNSQ > 2).

#### Overfit

The initial MFRM analysis demonstrated overfit for Item 11 (MNSQ < 0.5 and T > - 2) in weighted and unweighted fit but none in the sensitivity analysis.

**Table S2 here**

### Item separation reliability

The item separation reliability was 0.99, X^2^ =1958, df 23, P <0.000. This indicated the item difficulty estimates were significantly different from each other and covered a range of levels on the underlying scale.

### Rater and rating category analysis

Rater fit statistics revealed rater 2 showed severe misfit (weighted MNSQ = 4.7; T = 8.6) and rater 5 showed misfit (weighted MNSQ = 2.3; T = 2.3) (misfit indicated by weighted MNSQ > 2 and T > 2). As rater 5 analysed only 10/36 (28%) of the videos, the sparsity of data limited certainty in this result. Rater 6 was markedly more severe (severity estimate was 1.45 logits) than the remaining rater group, which had a narrow range in severity estimate from -0.81 to 0.13 logits. Rater separation reliability was 0.957, X^2^ = 228.20, df 5, P < 0.000, which confirmed the raters did not rate to a common standard with Rater 6 demonstrating the largest difference in comparison with the average (see Variable map, Figure 6 in the main article).

Rating category fit statistics revealed rating category 1 showed misfit with weighted MNSQ = 2.1 and T = 3.5 (weighted MNSQ > 2 and T > 2). Therefore, potential reasons for rating category 1 misfit were considered, including i) this rating category captured the partial demonstration of a recommended behaviour using the description ‘somewhat’ but this is not as clearly defined as ‘done’ or ‘not done’, which may lead to less consistent use, or ii) this rating category became inapplicable for items that described an educator behaviour, such as ‘the educator asked..’, that could only be done or not, not ‘partially done’.

The detailed rater fit and rater severity data are shown in Table S3.

The detailed rating category data are shown in Table S4.

**Table S3 here**

**Table S4 here**

### Variable map

The variable map is presented in Figure 6 (main article). The Rasch ‘ruler’ with the scale in logits (unit of measurement) is shown on the far left, representing the range of ‘feedback proficiency’. For each facet (items, raters and rating categories), the lowest levels with the lowest logit estimates are shown at the bottom of the variable map, with increasing levels towards the top of the variable map. Educators are shown on the left side of the left vertical hashed line, positioned according to their proficiency level (each ‘X’ represents 0.3 participant; this provides a slight distribution incorporating each participant’s estimate and standard error). Raters are shown in the middle section between the vertical hashed lines. Items are shown on the right side of the right vertical hashed line, located according to the average relative difficulty of the item (within each item, score categories (0,1,2) are distributed over a wider range, which is not shown here).

## S7 Exploratory factor analysis

### Principal components analysis

The data were found to be suitable for factor analysis (Bartlett’s test of sphericity was statistically significant with p = 0.000 and the Kaiser-Meyer-Olkin level was 0.825, exceeding the recommended value of 0.60).

Principal components analysis of the provisional instrument indicated that between three to five factors should be retained for further analysis (see Table S5 for the principal components analysis). This was based on the following: seven factors had eigenvalues over one, four to seven factors accounted for 54.8 to 69.0% total variance [17], parallel analysis revealed three factors had eigenvalues greater than corresponding criterion values for a randomly generated data set of the same size [19] (see Table S6 for the parallel analysis) and the scree plot displayed a sharp break point after the second factor [18] (Figure S1 for the scree plot).

Rotation using a direct oblimin approach was conducted on three-factor, four-factor and five-factor extractions. The four-factor solution with oblimin rotation showed a fairly simple structure: each factor had multiple items, each with high loadings on to it, which were theoretically aligned together, and no substantial loadings onto other factors; except for Item 5 that did not load onto any factor and Item 7 that crossloaded with similar substantial loadings onto two factors (see Table S7 for the principal components analysis with oblimin rotation). Communality values were low for items 5 and 25, indicating these items did not strongly share the same characteristics as the other items in their clusters. Rotation with a varimax approach gave a similar result. The four factors were named according to the highest loading items: factor one: *analyse performance*, factor two: *foster learner agency*, factor three: *plan improvement* and factor four: *set the scene*.

The four-factor solution was not perfect. Items 10 and 11 were both split between two factors and were not theoretically congruous with the other items clustered in each factor. However, in a five-factor solution with oblimin rotation, Items 10 and 11 strongly loaded together to form a fifth factor (See Figure 2 in main article for item list). Therefore, it was decided to create a fifth factor based on these items.

**Table S5 here**

**Table S6 here**

**Table S7 here**

**Figure S1 here**

## S8 Brief summary of qualitative study findings that informed refinements to the provisional instrument

The two qualitative analyses conducted on the feedback video data provided practical insights into ways that educators in our study worked with learners to foster their psychological safety and evaluative judgement. Below are brief summaries of the main findings of these studies that informed refinement of the provisional instrument.

Psychological safety: Thematic analysis of our feedback video transcripts explored ways psychological safety manifested during feedback sessions in clinical practice, published previously [20]. We inferred psychological safety when learners became increasingly candid and engaged in productive learning behaviours during the discussion, then we looked for corresponding educator behaviours. We created themes that described how educators had collaborated with learners to foster psychological safety. Based on our inductive analysis, we found educators seemed to foster psychologically safety by 1) setting expectations for learners to participate from the start; 2) collaborating with learners by demonstrating respect, compassion and assistance, and actively reducing the power gap; 3) focusing on continuing improvement, consistent with Dweck’s ‘growth mindset’ in which limitations and mistakes are expected during skill development and are seen as learning opportunities [21] and 4) valuing learners’ contributions to honest dialogue, by exploring learners’ perspectives, listening attentively and thoughtfully responding. These findings contributed to new items as well as revising items in the provisional instrument.

Evaluative Judgement: Thematic analysis of our feedback video transcripts explored what opportunities educators provided for learners to develop their evaluative judgement, published previously [22]. We reported three themes 1) *Educator’s invitations: I want to know what you think about the quality of your work*, which described how educators encouraged learners to share genuine evaluations of their work, often using follow-up probes for the learner to offer deeper reflections. 2) *Good work looks like this.* Educators commonly clarified standards, often using examples from their own practice. 3) *Calibration of the learner’s judgement through dialogue*. This theme highlighted how important it was educators to respond to learners’ comments about their work, to confirm or correct them, to fine tune their judgements.

Details of the item refinements, including new items, are outlined in detail in Appendix 1.

**Supplementary Figure legend**

Figure S1: Scree plot showing eigenvalues of consecutive extracted factors, from principal components analysis of the provisional instrument.

**Supplementary Tables legends**

Table S1: Frequency data for item ratings, resulting from administration of the provisional instrument to analyse feedback videos

Table S2: Item fit from multifaceted Rasch model analysis of the provisional instrument.

Table S3: Rater severity and rater fit from multifaceted Rasch model analysis of the provisional instrument, involving 6 raters ranked in order of severity (most severe rater at the top).

Table S4. Rating category results from multifaceted Rasch model analysis of the provisional instrument.

Table S5: Results of the principal components analysis of the provisional instrument.

Table S6. Comparison of eigenvalues from principal components analysis of the provisional instrument with criterion values from parallel analysis.

Table S7. Pattern and structure matrix for principal components analysis with oblimin rotation of a four-factor solution.

**References**

1. Pett M, Lackey NR, Sullivan JJ: **Designing and testing the instrument**. In: *Making sense of factor analysis: The use of factor analysis for instrument development in health care research.* edn. Thousand Oaks, California: SAGE Publications; 2003: 29-49.

2. Rasch G: **Probabilistic models for some intelligence and attainments tests.** Chicago: University of Chicago Press 1960.

3. Boone WJ: **Rasch Analysis for Instrument Development: Why, When, and How?** *CBE Life Sci Educ* 2016, **15**(4):rm4.

4. Granger C: **Rasch analysis is important to understand and use for measurement**. *Rasch Measurement Transactions* 2008, **21**(3):1122-1123.

5. Bond T, Fox CM: **Measuring facets beyond ability and difficulty**. In: *Applying the Rasch Model: Fundamental Measurement in the Human Sciences.* 3rd edn. London, United Kingdom: Taylor & Francis Group; 2015: 167-187.

6. Linacre JM: **Many-faceted Rasch measurement**, 2nd edn. Chicago: MESA Press; 1994.

7. Bond TG, Fox CM: **Applying the Rasch model: fundamental measurement in the human sciences**, 3rd edn. New York, NY: Routledge; 2015.

8. Boone WJ, Staver JR, Yale MS: **Multifaceted rasch measurement**. In: *Rasch Analysis in the Human Sciences.* edn. Dordrecht: Springer Netherlands; 2014: 423-448.

9. Wright BD, Linacre JM: **Reasonable mean-square fit values**. *Rasch Measurement Transactions* 1994, **8**(3):370.

10. Bond TG, Fox CM: **Rasch Model Requirements: Model Fit and Unidimensionality**. In: *Applying the Rasch Model : Fundamental Measurement in the Human Sciences.* edn. New York, NY: Taylor & Francis Group; 2015.

11. Linacre J: **Reliability, Separation and Strata: Percentage of Sample in Each Level**. *Rasch Measurement Transactions* 2013, **26**(4):1399.

12. Bartlett MS: **A note on the multiplying factors for various chi square approximations.** *Journal of the Royal Statistical Society* 1954, **16**:296-298.

13. Kaiser HF: **An index of factorial simplicity**. *Psychometrika* 1974, **39**(1):31-36.

14. Tabachnick BG, Fidell LS: **Using multivariate statistics**, 6th edn. Boston: Pearson Education; 2013.

15. Hutcheson GD, Sofroniou N: **The Multivariate Social Scientist: an Introduction to Generalized Linear Models**. London: Sage Publications; 1999.

16. Pallant JF: **Factor analysis**. In: *SPSS survival manual.* edn. Sydney: Allen & Unwin; 2016: 182-203.

17. Pett M, Lackey NR, Sullivan JJ: **Making Sense of Factor Analysis**. In*.* Thousand Oaks, California: SAGE publications; 2003.

18. Catell RB: **The scree test for number of factors.** *Multivariate Behavioral Research* 1966, **1**:245-276.

19. Horn JL: **A rationale and test for the number of factors in factor analysis.** *Psychometrika* 1965, **30**:179-185.

20. Johnson C, Keating J, Molloy E: **Psychological safety in feedback: What does it look like and how can educators work with learners to foster it?** *Medical Education* 2020, **54**(6):559-570.

21. Dweck CS, Yeager DS: **Mindsets: A View From Two Eras**. *Perspectives on Psychological Science* 2019, **14**(3):481-496.

22. Johnson C, Molloy E: **Building evaluative judgement through the process of feedback**. In: *Developing evaluative judgement in higher education Assessment for knowing and producing quality work.* edn. Edited by Boud D, Ajjawi R, Dawson P, Tai J. London: Routledge; 2018: 166-175.
