# Supplementary material for: Development of the Feedback Quality Instrument: a guide for health professional educators in fostering learner-centred discussions
Source: BMC Med Educ. 2021 Jul 12;21:382. doi: 10.1186/s12909-021-02722-8 (PMC8276464; doi:10.1186/s12909-021-02722-8)
Supplement: Supplementary file 2 — Additional file 2. [file 12909_2021_2722_MOESM2_ESM.docx]

**Tables for online supplementary information**

**Table S1: Frequency data for item ratings, resulting from administration of the provisional instrument to analyse feedback videos**

| **Item** | **Rating = 0** | **Rating = 1** | **Rating = 2** |
| --- | --- | --- | --- |
| **1** | 25 | 23 | 125 |
| **3** | 158 | 14 | 1 |
| **4** | 137 | 29 | 7 |
| **5** | 148 | 22 | 3 |
| **6** | 32 | 40 | 100 |
| **7** | 135 | 18 | 19 |
| **8** | 57 | 44 | 70 |
| **9** | 65 | 48 | 58 |
| **10** | 11 | 18 | 142 |
| **11** | 8 | 43 | 122 |
| **12** | 133 | 21 | 19 |
| **13** | 62 | 54 | 57 |
| **14** | 31 | 34 | 108 |
| **15** | 30 | 46 | 97 |
| **16** | 21 | 56 | 95 |
| **17** | 9 | 31 | 66 |
| **18** | 11 | 28 | 68 |
| **19** | 53 | 30 | 24 |
| **20** | 8 | 28 | 135 |
| **21** | 57 | 55 | 60 |
| **22** | 67 | 59 | 46 |
| **23** | 139 | 21 | 13 |
| **24** | 152 | 13 | 7 |
| **25** | 165 | 6 | 1 |

Rating scale: 0 = not seen; 1 = done somewhat or sometimes; 2 = done consistently

**Table S2: Item fit from multifaceted Rasch model analysis of the provisional instrument.**

**
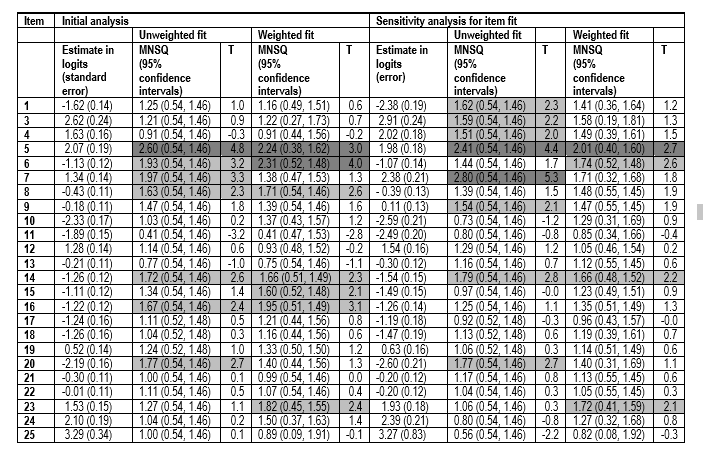
**

Shading: MNSQ = greater than 1.5, up to and including 2 and T = greater than 2; MNSQ = greater than 2 and T = greater than 2

Abbreviations: MNSQ = mean square of the residuals; T = T statistic (normalised equivalent).

**Table S3:** **Rater severity and rater fit from multifaceted Rasch model analysis of the provisional instrument, involving 6 raters ranked in order of severity (most severe rater at the top).**

|  |  | **Unweighted fit** | | **Weighted fit** | |
| --- | --- | --- | --- | --- | --- |
| **Rater** | **Severity estimate in logits**  (standard error) | **MNSQ**  (95% confidence intervals) | **T** | **MNSQ**  (95% confidence intervals) | **T** |
| **6** | 1.45 (0.09) | 2.50 (0.40, 1.60) | 3.6 | 1.88 (0.38, 1.62) | 2.3 |
| **5** | 0.13 (0.11) | 2.32 (0.12, 1.88) | 2.3 | 2.29 (0.13, 1.87) | 2.3 |
| **3** | -0.18 (0.06) | 1.72 (0.54, 1.46) | 2.6 | 1.67 (0.54, 1.46) | 2.4 |
| **4** | -0.19 (0.06) | 1.49 (0.54, 1.46) | 1.9 | 1.48 (0.54, 1.46) | 1.8 |
| **2** | -0.40 (0.06) | 4.68 (0.54, 1.46) | 8.6 | 4.67 (0.54, 1.46) | 8.6 |
| **1** | -0.81 (0.06) | 1.81 (0.53, 1.47) | 2.8 | 1.77 (0.53, 1.47) | 2.7 |

Abbreviations: MNSQ = mean square of the residuals; T = T statistic (normalised equivalent).

**Table S4. Rating category results from multifaceted Rasch model analysis of the provisional instrument.**

|  |  | **Unweighted fit** | | **Weighted fit** | |
| --- | --- | --- | --- | --- | --- |
| **Rating category** | **Category estimate in logits (error)** | **MNSQ**  **(95% confidence intervals)** | **T** | **MNSQ**  **(95% confidence intervals)** | T |
| **0** |  | 1.34 (0.54, 1.46) | 1.4 | 1.30 (0.53, 1.47) | 1.1 |
| **1** | -0.074 (0.049) | 2.19 (0.54, 1.46) | 3.9 | 2.06 (0.53, 1.47) | 3.5 |
| **2** | 0.074 | 1.23 (0.54, 1.46) | 1.0 | 1.21 (0.53, 1.47) | 0.8 |

Abbreviations: MNSQ = mean square of the residuals; T = T statistic (normalised equivalent).

**Table S5:** **Results of the principal components analysis of the provisional instrument.**

| **Total Variance Explained** | | | | | | | |
| --- | --- | --- | --- | --- | --- | --- | --- |
| Component | Initial Eigenvalues | | | Extraction sums of squared loadings | | | Rotation sums of squared loadings |
|  | Total | Percentage of variance | Cumulative percentage | Total | Percentage of variance | Cumulative percentage | Total |
| 1 | 7.60 | 31.7 | 31.7 | 7.60 | 31.7 | 31.7 | 5.5 |
| 2 | 2.58 | 10.7 | 42.4 | 2.58 | 10.7 | 42.4 | 4.8 |
| 3 | 1.54 | 6.4 | 48.8 | 1.54 | 6.4 | 48.8 | 3.8 |
| 4 | 1.44 | 6.0 | 54.8 | 1.44 | 6.0 | 54.8 | 1.8 |
| 5 | 1.27 | 5.3 | 60.1 | 1.27 | 5.3 | 60.1 | 2.3 |
| 6 | 1.13 | 4.7 | 64.8 | 1.13 | 4.7 | 64.8 | 1.8 |
| 7 | 1.01 | 4.2 | 69.0 | 1.01 | 4.2 | 69.0 | 1.3 |
| 8 | .88 | 3.7 | 72.7 |  |  |  |  |
| 9 | .80 | 3.3 | 76.0 |  |  |  |  |
| 10 | .74 | 3.1 | 79.1 |  |  |  |  |
| 11 | .65 | 2.7 | 81.8 |  |  |  |  |
| 12 | .60 | 2.5 | 84.3 |  |  |  |  |
| 13 | .53 | 2.2 | 86.5 |  |  |  |  |
| 14 | .46 | 1.9 | 88.4 |  |  |  |  |
| 15 | .41 | 1.7 | 90.1 |  |  |  |  |
| 16 | .40 | 1.7 | 91.8 |  |  |  |  |
| 17 | .37 | 1.5 | 93.3 |  |  |  |  |
| 18 | .33 | 1.4 | 94.7 |  |  |  |  |
| 19 | .30 | 1.3 | 96.0 |  |  |  |  |
| 20 | .27 | 1.1 | 97.1 |  |  |  |  |
| 21 | .20 | .9 | 97.9 |  |  |  |  |
| 22 | .18 | .8 | 98.7 |  |  |  |  |
| 23 | .17 | .7 | 99.4 |  |  |  |  |
| 24 | .15 | .6 | 100.0 |  |  |  |  |

**Table S6.** **Comparison of eigenvalues from principal components analysis of the provisional instrument with criterion values from parallel analysis.**

| Factor | Criterion value from parallel analysis | Actual eigenvalue from PCA | Decision |
| --- | --- | --- | --- |
| 1 | 1.7542 | 7.6 | **Accept** |
| 2 | 1.6181 | 2.57 | **Accept** |
| 3 | 1.5180 | 1.54 | **Accept** |
| 4 | 1.4380 | 1.436 | Reject |
| 5 | 1.3103 | 1.270 | Reject |

Abbreviation: PCA = principal components analysis

**Table S7. Pattern and structure matrix for principal components analysis with oblimin rotation of a four-factor solution.**

| **Item** | **Pattern coefficients** | | | | **Structure coefficients** | | | | **Communalities** |
| --- | --- | --- | --- | --- | --- | --- | --- | --- | --- |
|  | **Factor** | | | | **Factor** | | | |  |
|  | 1 | 2 | 3 | 4 | 1 | 2 | 3 | 4 |  |
| Item 1 | **.807** | .019 | -.015 | .005 | **.809** | .234 | .195 | .108 | .655 |
| Item 20 | **.798** | .051 | -.101 | -.075 | **.777** | .232 | .101 | .019 | .621 |
| Item 14 | **.777** | -.170 | .100 | .190 | **.781** | .091 | .292 | .275 | .672 |
| Item 15 | **.768** | -.113 | .193 | .044 | **.792** | .139 | .373 | .152 | .671 |
| Item 17 | **.705** | .115 | .041 | .153 | **.766** | .339 | .267 | .269 | .631 |
| Item 18 | **.665** | .225 | -.017 | .156 | **.741** | **.427** | .222 | .276 | .627 |
| Item 11 | **.627** | **.413** | -.173 | -.107 | **.680** | **.528** | .053 | .017 | .633 |
| Item 16 | **.614** | -.096 | .254 | .101 | **.665** | .137 | **.406** | .202 | .519 |
| Item 6 | .051 | **.797** | .032 | -.021 | .270 | **.813** | .202 | .127 | .665 |
| Item 8 | .081 | **.770** | .188 | -.007 | .335 | **.829** | .362 | .164 | .732 |
| Item 9 | .007 | **.709** | -.002 | .235 | .226 | **.751** | .179 | .357 | .617 |
| Item 19 | .077 | **.653** | .321 | -.111 | .319 | **.719** | **.454** | .060 | .633 |
| Item 13 | .358 | **.522** | .103 | .038 | **.530** | **.646** | .305 | .189 | .566 |
| Item 12 | -.116 | **.519** | .264 | .115 | .105 | **.561** | .357 | .230 | .400 |
| Item 10 | .366 | **.467** | -.148 | -.210 | **.427** | **.499** | .006 | -.106 | .411 |
| Item 23 | -.074 | .233 | **.727** | .146 | .192 | .385 | **.778** | .289 | .683 |
| Item 24 | -.116 | .234 | **.602** | .057 | .107 | .334 | **.629** | .175 | .455 |
| Item 22 | .264 | .128 | **.553** | -.108 | **.426** | .292 | **.630** | .033 | .495 |
| Item 21 | .186 | .132 | **.523** | .238 | .384 | .328 | **.634** | .365 | .533 |
| Item 25 | .067 | -.073 | **.474** | -.158 | .148 | .014 | **.452** | -.089 | .237 |
| Item 4 | .102 | .037 | -.065 | **.734** | .189 | .177 | .081 | **.743** | .566 |
| Item 3 | .160 | -.062 | -.006 | **.640** | .223 | .089 | .121 | **.649** | .445 |
| Item 7 | -.173 | **.465** | -.161 | **.566** | -.017 | **.483** | -.024 | **.599** | .571 |
| Item 5 | .098 | -.047 | .178 | .229 | .160 | .055 | .229 | .261 | .114 |

Footnote: Major loadings for each item in bold
