# Supplementary material for: Development of the Feedback Quality Instrument: a guide for health professional educators in fostering learner-centred discussions
Source: BMC Med Educ. 2021 Jul 12;21:382. doi: 10.1186/s12909-021-02722-8 (PMC8276464; doi:10.1186/s12909-021-02722-8)
Supplement: Supplementary file 3 — Additional file 3. [file 12909_2021_2722_MOESM3_ESM.docx]

**Figure for online supplementary information**

**Figure S1: Scree plot showing eigenvalues of consecutive extracted factors, from principal components analysis of the provisional instrument.**

**
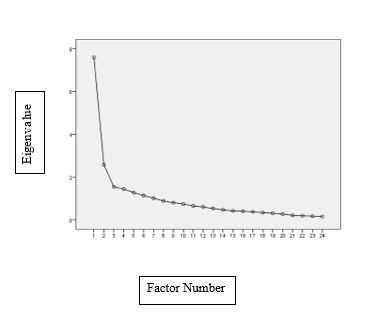
**
