# Supplementary material for: Development of the Feedback Quality Instrument: a guide for health professional educators in fostering learner-centred discussions
Source: BMC Med Educ. 2021 Jul 12;21:382. doi: 10.1186/s12909-021-02722-8 (PMC8276464; doi:10.1186/s12909-021-02722-8)
Supplement: Supplementary file 4 — Additional file 4. [file 12909_2021_2722_MOESM4_ESM.docx]

# Appendix 1: Detailed item revisions, with rationale, to the provisional feedback instrument

## The FQI evaluates the quality of an educator’s contribution during a face-to-face verbal feedback interaction related a learner’s observed performance in clinical practice.

## The ‘concept catcher’, in grey italics, encapsulates the essence of each item, to assist the user.

## Desirable criteria

**The instrument overall should:**

- be comprehensive (minimise gaps)

- be parsimonious (minimise overlap)

**Each item should**:

- be generally applicable during face-to-face feedback discussions

- target a single distinct attribute

- describe pertinent observable educator behaviour

- be unambiguous (description clear and simple)

- make sense with the three rating options

| **Provisional item** | **Item aim** | **Any proposed item changes and rationale, with results from Multifaceted Rasch model analysis (MFRM), exploratory factor analysis (EFA) and qualitative research on psychological safety and evaluative judgement.** | **Revised item** |
| --- | --- | --- | --- |
| *Item 1: Based on observed performance*  The educator’s comments were based on observed performance | Link the educator’s performance comments with specific example of relevant performance, so the learner can understand the basis. | • Combine items 1 & 17 into a single item, as they overlap.  • Remove ‘based’ as it may not be observable. For example, it could occur within the educator’s mind or could be satisfied by the context alone (such as workplace-based assessment).  • EFA: high loading [.807] with ‘analyse performance’ alone.  • MFRM: no weighted misfit. | *Specific instance*  The educator linked their comments to a neutral description of what the learner did (action, decision or behaviour) and the consequences. |
| *Item 2: Timely feedback*  The educator offered to discuss the performance as soon as practicable | Timely feedback. | • Deleted from the instrument during pilot testing, as it would occur before a feedback conversation. | Deleted |
| *Item 3: Feedback purpose clear*  The educator explained that the purpose of feedback is to help the learner improve their performance | Explicitly affirm the objective for the feedback conversation, to focus both participants | • Revise concept catcher, to make phrasing simpler and more similar to other concept catchers by using an active verb first.  • EFA: high loading [.640] with ‘set the scene’ alone  • MFRM: no weighted misfit. | *Clarify purpose*  The educator explained that the purpose of feedback is to help the learner improve their performance. |
| *Item 4:* *Establish a non-judgmental atmosphere: ‘here to help’*  The educator indicated that while developing a skill, it is expected that some aspects can be improved and the educator is here to help, not criticise | Foster a ‘learning orientation’ that focuses on continually developing skills. Mistakes or skill gaps are expected while learning a skill and the educator’s role is to assist the learner to develop their skills, not criticise.  This aligns with Dweck’s ‘growth mindset’ [1]. | • Revise the concept catcher. Remove ‘non-judgemental’, as judgement is an integral component of performance analysis, especially during summative assessments. New concept catcher highlights that mistakes are an expected part of learning and provide learning opportunities.  • In the item, more directly address learners’ fear regarding negative consequences if performance mistakes or omissions are exposed and highlight the associated learning opportunities.  • EFA: high loading [.734] with ‘set the scene’ alone  • MFRM: no weighted misfit. | *Expect mistakes and regard them as learning opportunities*  The educator conveyed the view that mistakes or omissions are expected while developing skills and are opportunities to continue refining skills. |
| *Item 5: Clarify feedback process, so learner knows what to expect*  The educator described the intended process for the feedback discussion | Create an opportunity for the learner to understand and contribute to expectations. The purpose is for the educator to increase transparency and share power over the session plans, in order to reduce excessive learner anxiety related to ‘not knowing what to expect’, which may interfere with attention, complex thinking and memory. | • Revise item by changing ‘process’ to ‘expectations’ and list key topics to consider, by clarifying the expectation to consider the learner’s input regarding the session plan by .  • EFA: does not load substantially onto any factor. Highest loading is with ‘set the scene’ [229] but very low communality [.114].  • MFRM: weighted misfit in original analysis (MNSQ =2.2, T =3.0) and sensitivity analysis (MNSQ =2.0, T =2.7)  Potential contributory factors  - Behaviour rarely seen: item rated as 0 = ‘not done’ in 148/174 (85%) sets of video analysis  - Phrasing vague. | *Discuss session plan, so learner knows what to expect*  The educator discussed expectations for the feedback session, taking the learner’s perspective into account.  (This may cover the session outline; time available; value of interactive dialogue and collaboration). |
| *Item 6: Encourage dialogue*  The educator encouraged the learner to engage in interactive discussions | • Promote interactive dialogue, with both participants contributing and building on each other’s comments, for the purpose of establishing a shared understanding and co-constructing ideas. | • Make item more explicit regarding what constitutes ‘interactive dialogue’ and add supplementary list of illustrative examples, based on parallel qualitative research on psychological safety.[2]  • EFA: high loading [.797] with ‘foster learner agency’ alone  • MFRM: weighted misfit in original analysis (MNSQ =2.3, T = 4.0) and sensitivity analyses, but less (MNSQ = 1.7, T = 2.6)  Potential contributory factors:  - Phrasing vague. | *Promote interactive dialogue*  The educator encouraged discussions in which both perspectives were shared and thoughtfully responded to.  (This may include agreeing, seeking more information, checking information, asking questions, building on an idea or contesting an idea with the rationale). |
| *Item 7: Seek learner’s priorities*  The educator asked the learner about their learning priorities for the observation and feedback discussion, and responded to them | • Find out about the learner’s learning priorities for the session. | • In the item, remove ‘observation’ as this instrument concerns the feedback discussion, not the prior observation of performance.  • Use ‘explore’ instead of ‘ask’, to emphasise that a single question may be insufficient, and the educator may need to make additional efforts to discover the learner’s priorities. This phrasing also makes sense with each rating category.  • Revise phrasing, so the meaning is more clear and simple.  • Keep item within ‘set the scene’ as it is important to find out a learner’s priorities at the start.  • EFA: cross loading on two factors: ‘set the scene’ [.566] and ‘foster learner agency’ [.465]. This makes sense as this item contributes to both.  • MFRM: no weighted misfit.  • Middle rating category (1= done somewhat or sometimes) does not make sense with ‘ask’, which results in a dichotomous outcome (educator did/did not ask). | *Seek learner’s priorities*  The educator explored what the learner most wanted to raise in the feedback session. |
| *Item 8: Encourage learner to ‘work it out for themselves’*  The educator encouraged the learner to consider the issues and possible solutions during the feedback discussion. | • Promote active learning by supporting learner to reflect and problem solve, aligned to a social constructivist paradigm. | • In the concept catcher, change ‘encourage’ to ‘support’, to avoid duplication.  • EFA: high loading [.770] with  ‘foster learner agency’ alone  • MFRM: weighted misfit in original analysis (MNSQ =1.7, T =2.6) but not the sensitivity analysis (MNSQ =1.5, T =1.9). | *Support learner to ‘work it out for themselves’*  The educator encouraged the learner to consider the issues and possible solutions during the feedback discussion. |
| *Item 9:* *Encourage learner to focus on learning, rather than trying to cover up limitations*  The educator encouraged the learner to discuss difficulties and ask questions regarding the performance so the educator could help the learner to develop solutions. | • Deliberate focus on encouraging learning behaviours, in particular ‘what was difficult?’ and ‘any questions?’ , in order to offer assistance, in line with encouraging a ‘Growth mindset’ [1]. | • No change.  • EFA: high loading [.709] with ‘foster learner agency’ alone  • MFRM: no weighted misfit. | No change. |
| *Item 10:* *Acknowledge learner’s emotional response*  The educator acknowledged and responded appropriately to emotions expressed by the learner. | • Respond appropriately to learner’s emotional reaction during a feedback discussion. Emotions are commonly stimulated and if not attended to, they can consume attention and hence hinder reasoning, learning and memory. | • In the concept catcher, use ‘attend’ (i.e. ‘pay attention to’), as this best captures the intention of the item (whereas the item must be strictly observable).  • In the item, remove ‘acknowledged’, as this is included within ‘respond’.  • EFA: In a 5-factor solution with oblimin rotation, high loading [.735] with ‘foster psychological safety’ and Communality .68  • MFRM: no weighted misfit | *Attend to learner’s emotions*  The educator responded appropriately to emotions expressed by the learner. |
| *Item 11:* *‘Best interests at heart’*  The educator showed respect and support for the learner. | • Demonstrate respect and support for the learner, with the aim of working with the learner to foster a psychologically safe learning environment that encourages learning behaviours and intrinsic motivation (which requires sentiments of autonomy, relatedness and competence, in line with self-determination theory). | • Replace this with new additional items that describe specific educator behaviours to demonstrate respect and support.  • Item is broad and does not describe specific observable behaviours.  • EFA: In a 5-factor solution with oblimin rotation, high loading [.607] with ‘foster psychological safety’ and Communality .73  • MFRM: no weighted misfit. | Replaced by new additional items. |
| *Item 12:* *Clarify the value of self-assessment*  The educator asked what the learner understood about the benefits of self-assessment and helped clarify. | • Promote the value of a thorough learner self-assessment: to practise evaluative judgement and to reveal their understanding of the target performance and provide an opportunity for learner to proffer their learning needs. | • Revise item and use ‘promoted’’ instead of ‘ask, as ‘ask’ can be ‘one off’ and superficial. This also makes sense with each rating category.  • Revise concept catcher and use ‘advocate for’ instead of ‘clarify’  • Add supplementary information to explain the value of self-assessment, to assist the educator, informed by the qualitative analysis on evaluative judgement.  • EFA: moderately high loading [.519] with ‘foster learner agency’ alone  • MFRM: no weighted misfit  • Middle rating category (1= done somewhat or sometimes) does not make sense with ‘ask’, which results in a dichotomous outcome (educator did/did not ask).  • Behaviour rarely seen: item rated as ‘not done’ in 133/173 (77%) sets of video analysis data. | *Advocate for the value of* *self-assessment*  The educator promoted the benefits of self-assessment in discussion with the learner.  (Self-assessment provides opportunity for a learner to describe key features of the target performance, analyse their own performance in comparison of the target performance and raise learning needs; this involves practising valuable learning skills such as reflection, evaluative judgement and self-regulated learning). |
| *Item 13:* *Learner self-assessment*  The educator asked the learner to identify key similarities and differences between the learner’s performance and the target performance. | • Thorough self-assessment by the learner, involving a comparison of the learner’s performance with the target performance. | • In the item, use ‘encouraged’ instead of ‘asked’, to emphasise that a single question is often insufficient, in which case the educator may need to make additional efforts to enable the learner to voice their self-assessment, informed by the qualitative analysis on evaluative judgement.  This also makes sense with each rating category.  • Position with ‘Analyse performance’ to emphasise inclusion of the learner’s self- assessment.  • EFA: moderately high loading with ‘foster learner agency’ [.522] and lower loading with ‘analyse performance’ [.358]  • MFRM: no weighted misfit  • Middle rating category (1= done somewhat or sometimes) does not make sense with ‘ask’, which results in a dichotomous outcome (educator did/did not ask). | *Learner self-assessment*  The educator encouraged the learner to identify key similarities and differences between the learner’s performance and the target performance. |
| *Item 14:* *Target performance and reasoning clear*  The educator clarified with the learner key features of the target performance and explained the reasoning. | • Explain what the task ‘should look like’ and the rationale, to strengthen the learner’s understanding of the target performance, particularly in a specific context | • No change, supported by the qualitative analysis on evaluative judgement.  • EFA: high loading within performance analysis alone [.777]  • MFRM: mild weighted misfit in original analysis (MNSQ =1.7, T =2.3) and sensitivity analysis (MNSQ =1.7, T =2.2)  No problems apparent on review. | No change |
| *Item 15:* *Educator assessment, including clear performance gap*  The educator clarified with the learner similarities and differences between the learner’s performance and the target performance. | • Comparison of the learner’s performance with the target performance, to clarify i) which aspects were done effectively, thereby promoting feelings of competence and reward for the effort put into skill development; and ii) which aspects were not done effectively, thereby clarifying performance gap, which focuses attention and motivates change.  • Typically, this is most useful when focused on the learner’s zone of proximal development [3]. | • In the concept catcher, use ‘analysis’ instead of ‘assessment’, to focus on the comparison of the learner’s performance with the target performance, in line with a learning orientation, as opposed to ‘assessment’, which could imply just a ‘score’ or a judgement that the performance was ‘good enough’ (or not).  • Add supplementary recommendation to consider focusing on the zone of proximal development, to enhance developmental focus.  • EFA: high loading [.768] with ‘analyse performance’ alone  • MFRM: minor weighted misfit in original analysis (MNSQ =1.6, T =2.1) but not in the sensitivity analysis (MNSQ =1.2, T =0.9). | *Educator’s performance analysis*  The educator clarified with the learner similarities and differences between the learner’s performance and the target performance.  (Consider focusing on the ‘development zone’ i.e. around the learner’s current position on the learning curve). |
| *Item 16:* *Educator comments on a few, important issues*  The educator’s comments focused on key issues for improving the performance | • Prioritise a few comments and avoid numerous or low value comments which contribute to cognitive overload. Select topics which are important and most likely to improve performance. | • Shorten the concept catcher, to make phrasing simpler and add ‘prioritise’ for emphasis.  • In the item, add ‘a few’ as an important observable feature and change ‘issues’ to points’ as ‘issues’ may imply ‘problems’.  • Add a supplementary recommendation to consider improvements beyond just the current task, to assist the educator.  • EFA: high loading [.614] with ‘Analyse performance’ alone.  • MFRM: weighted misfit in original analysis (MNSQ =1.95, T =3.1) but not in the sensitivity analysis (MNSQ =1.4, T =1.3). | *Prioritise comments*  The educator’s comments focused on a few, key points for improving performance.  (This may focus on improving the current task or broader learning strategies). |
| *Item 17:Specific instance (‘what happened’)*  First the educator described, using neutral language, what the learner did (action, decision or behaviour), and the consequences | • Items 17-19 were originally designed to address corrective comments (only).  • Link educator’s comments with specific example/s of relevant performance, so the learner can understand the basis.  • Use neutral language when describing the performance (i.e. avoid critical language), to isolate ‘observed performance’ from the educator’s interpretation or evaluation. | • Combine items 1 & 17 into a single item, as they overlap.  • EFA: high loading [.705] with ‘analyse performance’ alone  • MFRM: no weighted misfit.  • Item not ‘generally applicable’ (items 17-19 were originally designed to address corrective comments only). | See revised Item 1 |
| Item 18: *Educator’s perspective clear (‘why it matters’)*  The educator clearly explained their perspective on the learner’s actions, including the reason for their concern | • Items 17-19 were originally designed to address corrective comments (only).  • Clear explanation of the educator’s comments about the learner’s performance (information or their opinion), accompanied by the rationale. This assists the learner’s understanding of the educator’s perspective. It also supports transparency and autonomy by providing substantiation, to allow the learner to judge the validity for themselves | • Broaden the item to apply whenever the educator provides information or their opinion, so it is ‘generally applicable’.  • Item not ‘generally applicable’ (items 17-19 were originally designed to address corrective comments only).  • EFA: high loading [.665] with ‘Analyse performance’ alone.  • MFRM: no weighted misfit. | *Educator’s perspective clear*  The educator’s comments (information or opinion), accompanied by the reasoning, were clearly explained. |
| *Item 19: Educator explores learner’s perspective (‘why’ learner acted as they did)*  The educator explored the learner’s perspective and reasoning to reveal the basis for the learner’s actions (e.g. what was the learner trying to do and options considered/ difficulties encountered) | • Items 17-19 were originally designed to address corrective comments (only).  • Important for the educator to explore and understand the learner’s perspective, particularly the drivers for the learner’s actions, so the correct learning needs can be targeted | • Broaden the item, to highlight the value of exploring and listening attentively to the learner’s perspective at all times, so the item is ‘generally applicable’. This shows respect and humility by valuing the learner’s perspective (showing interest & willing to be influenced by it).  • Position item within ‘Foster psychological safety’ as it fits best there, once generalised.  • EFA: high [.635] loading with ‘Foster learner agency’.  • MFRM: no weighted misfit.  • Item not ‘generally applicable’ (items 17-19 were originally designed to address corrective comments only). | *Value learner’s perspective*  The educator explored the learner’s perspective and reasoning, and demonstrated attentive listening. |
| *Item 20:* *Focus on actions, not the person (‘did’ not ‘is’)*  The educator’s comments were focused on the learner’s actions not personal characteristics | • Focus the learner’s attention on improving task performance and avoiding attention on ‘self’, which risks strong emotional reactions concerning innate characteristics [4]. | • No change  • EFA: high loading [.798] with ‘Analyse performance’ alone.  • MFRM: no weighted misfit. | No change |
| *Item 21:* *Select learning priorities: most useful (important and relevant) for the learner*  The educator helped the learner to select a couple of key aspects of the performance to improve | • Educator and learner deliberately select 1-2 learning goals that are most useful for the learner, in order to improve performance. The learner’s involvement is important to support autonomy and motivation. | • In the item, remove ‘the’ from ‘the performance’ to expand the focus to include both the current task and broader learning strategies.  • Change ‘a couple of’ to ‘few’ for to make the phrasing simpler.  • Add supplementary information to recommend potentially valuable options.  • EFA: moderately high loading [.523] with ‘Plan improvements’ alone  • MFRM: no weighted misfit. | *Select learning priorities: most useful (important and relevant) for the learner*  The educator helped the learner to select a few, key aspects of performance to improve  (Consider learner’s request; key mistake or omission or ‘next steps’). |
| *Item 22:* *Develop the action plan: how to do it!*  The educator helped the learner to work out how they could improve their performance and specify the practical steps to achieve it. | • Educator and learner develop specific and practical strategies to improve performance. | • Revise concept catcher and item, to make phrasing simpler.  • Expand improvement focus to include both the current task and broader learning strategies.  • EFA: moderately high loading with [.553] ‘Plan improvements’ alone  • MFRM: no weighted misfit. | Develop the action plan  The educator helped the learner to develop specific and practical plans to improve their performance.  (The plans may relate to the current task and/or broader learning strategies). |
| *Item 23:* *Check the learner understands the plans*  The educator checked if the learner understood their learning goals and action plan by asking them to summarise it in their own words | • Check the learner’s understanding of the learning goals and action plan, using a teach-back technique [5]. | • Combine items 23 & 24 to reduce overlap.  • Shorten the concept catcher, to make phrasing simpler.    • EFA: high loading [.727] with ‘Plan improvements’ alone.  • MFRM: mild weighted misfit in original analysis (MNSQ =1.8, T =2.4) and less in sensitivity analysis (MNSQ =1.7, T =2.1).  • Behaviour rarely seen: rated as ‘not done’ in 139/173 (80%) sets of video analysis. | Check plans and rationale understood  The educator checked if the learner understood their learning goals and action plan, accompanied by the rationale, by asking them to summarise it in their own words. |
| *Item 24:* *Checks the learner understands the rationale: ‘why it’s better’*  The educator checked if the learner understood the rationale for their learning goals and action plan | • Check the learner’s understanding of the rationale underpinning the learning goals and action plan, using a teach-back technique [5]. | • Combine items 23 & 24 to reduce overlap.  • EFA: high loading [.602] with ‘Plan improvements’ alone.  • Rasch: no weighted misfit. | Combined with item above |
| *Item 25:* *Plan opportunities to review the impact of the feedback*  The educator discussed with the learner possible subsequent opportunities for the learner to review their progress | • Plan how to review progress and whether the action plan resulted in improved performance, to complete the feedback loop. Subsequent performance may be reviewed by another clinician. | • Shorten the concept catcher, to make phrasing simpler.  • Remove ‘subsequent’ as unnecessary, to make phrasing simpler.  • EFA: low loading [.474] with ‘Plan improvements’ and communality low at .237.  • MFRM: no weighted misfit.  • Behaviour very rarely seen: item rated as ‘not done’ in 165/172 (96%) sets of video analysis data. | *Plan how to review progress*  The educator discussed with the learner possible opportunities for the learner to review their progress. |
| New items to expand domain ‘foster psychological safety’ based on the qualitative analysis[2] | | | |
| New item | • Educator demonstrate respect, humility and inclusiveness by appreciating the learner’s contributions, attributes, skills and potential. |  | *Appreciate learner’s contributions*  The educator expressed appreciation for the learner’s contributions.  (This may include the learner’s input into the discussion; learner’s contribution to healthcare practice; learner’s attributes, skills, or future potential). |
| New item | • Educator demonstrate respect for learner’s autonomy |  | *Respect learner’s autonomy*  The educator showed respect for the learner’s autonomy.  (This may include encouraging the learner to take a turn to lead the conversation; state their opinion or preference; make a choice; or contest the educator’s comments for the purpose of learning). |
| New item | • Educator demonstrate support and care by expressing compassion for difficulties experienced by the learner. |  | *Show compassion*  The educator expressed compassion for difficulties experienced during training, raised by the learner |
| New item | • Educator demonstrate humility by recognising own limitations |  | *Show humility and recognise own limitations*  The educator conveyed the view that everyone has limitations, including themselves.  (This may include acknowledging educator’s limitations e.g. routine uncertainty during clinical practice; beyond their speciality; educator’s evaluation, opinion or advice contestable; made mistakes themselves while learning; or general limitations e.g. ‘always more to learn’ or ‘a common mistake’). |

References:

1. Dweck CS: **Motivational processes affecting learning**. *American Psychologist* 1986, **41**.

2. Johnson C, Keating J, Molloy E: **Psychological safety in feedback: What does it look like and how can educators work with learners to foster it?** *Medical Education* 2020, **54**(6):559-570.

3. Morris C, Blaney D: **Work-based learning** In: *Understanding medical education: evidence, theory and practice.* 2nd edn. Edited by Swanwick T. Oxford: The Association for the study of Medical Education 2014: 97-109.

4. Kluger AN, DeNisi A: **The effects of feedback interventions on performance: a historical review, a meta-analysis, and a preliminary feedback intervention theory**. *Psychological Bulletin* 1996, **119**(2):254-284.

5. Yen PH, Leasure AR: **Use and Effectiveness of the Teach-Back Method in Patient Education and Health Outcomes**. *Federal Practitioner* 2019, **36**(6):284-289.
